# Healthy, nudged, and wise: Experimental evidence on the role of information salience in reducing tobacco intake

**DOI:** 10.1002/hec.4509

**Published:** 2022-03-28

**Authors:** Adnan M. S. Fakir, Tushar Bharati

**Affiliations:** ^1^ Department of Economics University of Sussex Business School Brighton UK; ^2^ Department of Economics University of Western Australia Business School Perth Western Australia Australia

**Keywords:** Bangladesh, field experiment, health decision‐making, nudge, smoking, tobacco

## Abstract

We evaluate the performance of two behavioral interventions aimed at reducing tobacco consumption in an ultra‐poor rural region of Bangladesh, where conventional methods like taxes and warning labels are infeasible. The first intervention asked participants to daily log their tobacco consumption expenditure. The second intervention placed two graphic posters with warnings about the harmful effects of tobacco consumption on tobacco users and their children in the sleeping quarters of the participating households. While both interventions reduced household tobacco consumption expenditure, male participants who logged their expenditure substituted cigarettes with cheaper smokeless tobacco. The reduction in tobacco intake is larger among males with a non‐tobacco consuming spouse. Exploratory analysis reveals that risk‐averse males who spent relatively more on tobacco responded more to the logbook intervention. More educated, patient males with children below age five responded better to the poster intervention. The findings suggest that in countries with multi‐tiered tobacco excise tax structures, which incentivize downward substitution, extending complementary demand‐side policies that worked elsewhere to the rural poor might be unwise. Instead, policies may leverage something as universal as parental concern for their children's health to promote better health decision‐making.

## 1 INTRODUCTION

The rise in healthcare spending worldwide has raised concerns regarding its sustainability (Böhm et al., 2021; World Bank, 2018). While the rising needs of an aging population and costly technological improvements are some unavoidable driving factors, the burden of preventable lifestyle diseases forms a significant portion of these costs (Amadeo, 2021; Aon, 2018; Stanaway et al., 2018). Take smoking, for example, the behavior we examine in this study. Second only to high systolic blood pressure in its burden, smoking accounted for around 13% of all deaths and a little over 7% of disability‐adjusted life‐years lost in 2017 (Stanaway et al., 2018). Finding policy solutions to persuade people to make better health choices is crucial to ensuring a healthier future for all.

Economists and policy‐makers have long favored price regulation, through taxes and subsidies, as the go‐to policy tool to improve individual decision‐making. Tobacco is currently one of the highest‐taxed commodities across the world (WHO, 2018a, 2018b). The approach is rooted in sound economic theory. In some cases, like with taxes on cigarettes, it also receives overwhelming support from the voters and generates significant revenues (see Chaloupka and Warner (2000) for an interesting discussion of cigarette taxes). But price regulations have their share of criticisms for a variety of reasons.^1^ Besides, the health burdens of risky behavior, like smoking, are rapidly moving from high‐income to middle‐ and low‐income countries (Hammond, 2009; Jha & Peto, 2014; Mathers & Loncar, 2006) where multi‐tiered excise tax structure on tobacco can incentivize downward substitution from costlier to cheaper tobacco (Nargis, Hussain, et al., 2019; Ohsfeldt et al., 1997). In Bangladesh, the country we focus on, such substitutions have led to an increase in tobacco consumption (Nargis, Stoklosa, et al., 2019). Further, corruption and relatively strong corporate lobbies in these countries make effective tax policies difficult to enact and enforce (Rahman et al., 2019). Together, these factors necessitate the need for complementary approaches to reduce tobacco intake.

In recent years, policy interventions based on insights from behavioral economics have gained traction among scholars and legislators alike.^2^ The approach uses “nudges,” like providing information or increasing the relative convenience of engaging in healthy behavior, that seek to steer consumers towards desirable behaviors without limiting their freedom of choice (Camerer et al., 2003; Thaler & Sunstein, 2003). Examples include providing commitment devices to encourage savings, nutritional information labels on packaged food, calorie information on restaurant menus, and graphical warning labels on cigarette packs. The approach is not without its drawbacks and criticisms. There are concerns about the effectiveness and coherence of the nudges approach (Bonell et al., 2011; Marteau et al., 2011). Loewenstein et al. (2012) argue its conceptual appeal is so persuasive that it sometimes gets a pass with no or contrary empirical evidence. Others have found that soft nudges, especially providing information alone, are often ineffective and can also trigger perverse substitution behavior (Downs et al., 2009; Dupas & Miguel, 2017; Elbel et al., 2009; Kremer et al., 2019; Loewenstein, 2011; Monárrez‐Espino et al., 2014; Wisdom et al., 2010). Despite this, behavioral interventions are becoming a staple across countries (Christiano & Spring, 2017; Hussam et al., 2019; Macours & Vakis, 2014; Monárrez‐Espino et al., 2014; Ngo et al., 2018). In developing countries with multiple constraints on the effective use of tax instruments, it is even more important to accumulate evidence on the advantages and disadvantages of complementary behavioral interventions to improve health decision‐making.

Our aim with this study is to add fresh evidence on the effectiveness and limitations of behavioral interventions aimed at promoting smoking cessation in extremely poor parts of developing countries. We use a randomized field experiment to examine the impact of two behavioral interventions that improve the salience of information about the current or future cost of tobacco consumption among a rural poor population in the riverine islands (*chars*) of Gaibandha district in Northern Bangladesh. The first intervention asked participants to maintain a daily record of household expenditure on tobacco products (henceforth, the *Logbook intervention (LT)*). The intervention aimed to increase the salience of the immediate monetary cost (Thaler & Sunstein, 2009). By reminding participants of the cumulative expenditure on tobacco consumption, it also aimed at tackling the specific behavioral bias referred to as the “peanuts effect”, where people tend to pay less attention to small repeated payments than a single large payment (Weber & Chapman, 2005).

The second intervention placed two graphic warning posters (henceforth, the *Poster intervention (PT)*) in the sleeping quarters of the participating households. It aimed to remind participants constantly of the distant non‐pecuniary costs of tobacco consumption for them and their children. While similar to graphic warning labels on cigarette packs, it also depicts the harmful effect of tobacco consumption on a user's children. Since most participants were aware of the harmful effects of tobacco consumption at the baseline, the PT made the information about the health costs more salient. The two interventions did not provide any monetary incentives for participation, but we informed all participants in the treatment and control groups of their respiratory health status measured at baseline and end line. We also provided a record‐keeping logbook to each logbook‐intervention participant and two posters to each poster‐intervention participating household.

Using a 2‐stage clustered randomization design, we target 985 households from 24 *chars*, split roughly equally among the control, the logbook, and the PT groups. The sample is representative of the population living in the broader *char* region of Gaibandha district. Both the logbook and poster interventions significantly lowered overall tobacco consumption expenditure by 22.8% and 25.3% respectively, relative to the control group, in a 6‐week follow‐up. We find different effects for male and female participants. While both the logbook and poster interventions significantly lowered breath carbon‐monoxide (CO) levels for male participants by about 10% and 8.7%, respectively, male participants in the LT group substituted away from cigarettes to cheaper smokeless tobacco (SLT) products. There is no effect on *bidi* ^3^ consumption. Their expenditure on SLT increased by 22% relative to the control group. Consumption of SLT products increases the risk of oral or oropharyngeal cancer (Hoffmann & Djordjevic, 1997; Roosaar et al., 2008).^4^ The LT, that made the expenditure on tobacco more salient, pushed male participants to seek cheaper alternatives than to reduce tobacco consumption. We do not find any such substitution for the PT. Female participants, none of whom smoked, decreased their SLT consumption expenditure by about 10.7% due to the PT, but were unaffected by the LT. The results remain consistent after adjusting for the probability of false rejections due to multiplicity of tests using family‐wise error rate (FWER) *p*‐values, for the small number of clusters in the experiment using wild bootstrap‐*t* clustering method (Cameron et al., 2008), and to randomization inference (RI) permutation tests (Young, 2019).

Heterogeneity analysis shows that male participants with spouses who did not consume tobacco reduced their smoking tobacco consumption more in both the logbook and PT groups, indicative of possible treatment reinforcement by partners (Monden et al., 2003; vanDellen et al., 2016). We do not find a similar effect for female participants with non‐user spouses. Next, we conduct exploratory analysis to examine potential mechanisms by baseline participant characteristics. We find male participants in the LT group who were more risk‐averse and spent a higher percentage of total household expenditure on tobacco products are more likely to decrease their expenditure, but by switching to cheaper SLT products. Consistent with Becker and Murphy (1988)'s finding that stressful events can lead to addictive behavior, we find that males with fewer lifetime *char* relocations (fewer exposures to repeated shocks) decrease their consumption more. Male participants in the PT group who were more educated, more patient, or had children below the age of five lowered their tobacco intake more.

The findings provide important policy lessons. Curbing tobacco consumption and the associated health costs, especially in rural regions, remains a major challenge in developing countries.^5^ Expenditure on tobacco also crowds out household expenditure on education, housing, and clothing in Bangladesh (Husain et al., 2018). The markets in developed and developing countries vary along numerous dimensions and it is unwise to design interventions based on findings from developed countries. With poor tax infrastructure, strong lobbying by tobacco companies, and increasing affordability of alternatives, approaches like multi‐tiered excise tax structures are unlikely to succeed in extremely poor regions like Gaibandha (Barkat et al., 2012; Nargis, Stoklosa, et al., 2019). Complementary interventions targeting the demand side are called for.

However, as our findings point out, the effects of such interventions may not always be as straightforward as they may intuitively appear. Previous studies have cautioned that some types of interventions can trigger substitution from cigarettes to cheaper SLT, especially in settings with multi‐tiered excise tax structures (Nargis, Hussain, et al., 2019; Ohsfeldt et al., 1997). But behavioral interventions often overlook these possibilities. For example, we could not find any studies that examine the impact of cigarette packaging graphic warning labels on the consumption of SLT or *bidis*, given that the latter two seldom come with a graphical warning on their packaging.^6^ The first lesson, we believe, is to understand the need for carefully designing these interventions, grounding them firmly in theory so that such externalities can be predicted and corrected. A body of evidence around the effectiveness and consequences of different interventions in developing countries must be amassed. This study hopes to take a step in that direction. A second, more encouraging finding is that while information about the health costs may not be enough by itself, health advocacy campaigns can creatively leverage people's intrinsic concern for their children to reduce tobacco consumption and improve health for all. Our findings also suggest that, with most participants already aware of the health costs of tobacco consumption, it is likely the salience of the information through repeated exposure (via the nudges), and not the information itself, that induced the behavioral change.

Broadly, we also contribute to the growing literature on the causal impact of behavioral interventions in promoting health behavior (Cawley & Price, 2013; Jepson et al., 2010; List & Samek, 2015; Malotte et al., 1998; Mwabu, 2007; Volpp et al., 2008, 2009). The paper adds to the growing body of literature on addictive behavior, especially that on curbing tobacco intake using behavioral interventions (Cantrell et al., 2013; Fong et al., 2009; Karinagannanavar et al., 2011; Kuehnle, 2019; Monárrez‐Espino et al., 2014; Noar, Francis, et al., 2016; Noar, Hall, et al., 2016; Thrasher et al., 2011). To the best of our knowledge, this is the first study to document that even salience of the information about expenditure on harmful behavior can, through substitution, generate perverse effects.^7^ We are also the first to show that an intervention that increases the salience of the information about distant health costs is enough to motivate apparently present‐biased individuals to change their behavior if it leverages their concern for their children's health.

## 2 CONTEXT: STUDY SITE

Our experiment site is the *chars* (riverine islands) of rural Gaibandha district in northern Bangladesh. *Chars* are small landmasses formed along the *Jamuna* river (lower stream of the *Brahmaputra* river) from deposits of alluvium and silt (Sarker et al., 2020). The silt depositions make the *chars* extremely fertile (Sarker et al., 2003). However, living in the *chars* is immensely volatile because of frequent flooding, riverbank erosion, and thunderstorms (Howell, 2003; Sarker et al., 2015). Settlers who move to these precarious islands are often landless trying to escape abject poverty, and the majority opt to be farmers (Poncelet et al., 2010).

Connection to the outside world is only through waterways. There are two boat docking points (*ghats*) connecting mainland Gaibandha to the *chars*. Public boats are available between 8 a.m. and 8 p.m. that complete three to five round‐trips per day. Travel outside these hours must be by private boats at a premium price. There is no electricity and poor cellphone coverage in the *chars*, limiting communication and access to one‐way or two‐way media, like television, radio, or social media platforms. As a result, people are rarely exposed to national anti‐tobacco campaigns. The remoteness of the *chars* also results in limited development activities by non‐governmental organizations (NGOs) in the region. There were no active anti‐tobacco campaigns in the month prior to the baseline survey. All these factors make the *chars* extremely secluded, with poor access to health care and education (Kabir, 2006).

At baseline, participating households spent about 9% of total household expenditure (sample mean) on tobacco products. This is in a region where most households live below the poverty line of $1.90/day. With the *char*‐dwellers living in families, this figure increases to almost 39% of per capita household expenditure. Alarming as it is, the information is not new. Efroymson et al. (2001, p.214), in a study on the opportunity costs of tobacco use for the poor in Bangladesh, state that “the poorest households spend half as much on tobacco as on health, and almost 10 times as much on tobacco as on education.” Tobacco consumption is rooted in the local *char* culture. Khair et al. (2020), in a qualitative study, posits that the high tobacco dependency is a symptom of larger socio‐economic problems. Several factors contribute to the “culture” of tobacco dependency in the *chars*. First, even though the legal framework prohibits the promotion of tobacco products (NTCC, 2013), campaigns by the tobacco industry in the form of free samples, discounts, coupons, or free branded clothing, are frequent. Tobacco companies also distribute cheaper varieties for the *chars* that are often unavailable in mainland Gaibandha.^8^

Second, similar to other settings, tobacco facilitates socialization and the exchange of social capital in the *chars* (Cutler & Glaeser, 2010). Tobacco is offered to guests as a symbol of hospitality. Agriculture employers also provide tobacco for free to hired labor. Laborers, who often work on water‐logged land, believe that “smoking increases body temperature and therefore productivity” (Khair et al., 2020, p.7). This further motivates their tobacco consumption. Boys treat smoking as “a ritual to manhood”, and may sometimes engage in it to avoid social penalty due to not conforming with peers (Khair et al., 2020, p.5). The smoking rates among men are almost 80% compared to the national average of 46.0% (Marquez et al., 2019). The average male participant reported having started smoking by the age of 14, with some starting as early as 8 by imitating the elders at home. It is a cultural taboo for females of the *chars* to smoke. Instead, they consume SLT products, such as *zarda* and *gul*,^9^ which, like in the case of males, facilitate social interactions and exchanges. Females report a different motivation for their SLT consumption. In the absence of access to doctors, they resort to SLT seeking temporary relief from gum pain, headaches, and other ailments.

**FIGURE A1:**
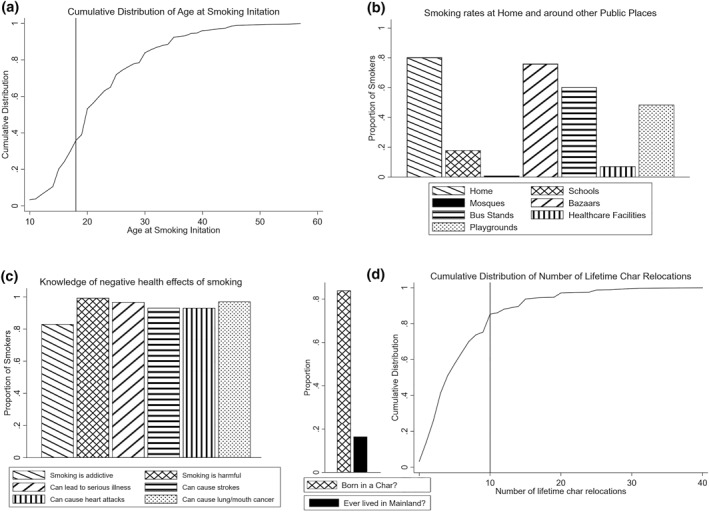
Selected information about tobacco consumers of the *chars*. *Notes*: Figures based on information from 827 male smokers. Panel (a) provides a cumulative distribution of age of smoking initiation. Panel (b) reports the proportion of smokers who smoke at public places, including at home. Panel (c) shows the share of participants who know of the negative health effects of smoking. Panel (d) reports the sample share born in the *chars* and the distribution of lifetime *char* relocations

Third, the psychological stress of living in the *chars*, with frequent flooding and riverbank erosion, imbue a sense of constant stress, uncertainty, and risk. Studies have previously shown stress is correlated with higher tobacco consumption (De Vogli & Santinello, 2005; Islam & Walton, 2019). Consistent with it, many men in the *chars* report using smoking as a mechanism to cope with the daily stress and anxiety (Fidler & West, 2009; Robles et al., 2017). Stress and uncertainty are recurring themes for many *char*‐dwellers. Around 20% of households reported having had at least 10 lifetime *char* relocations within Gaibandha at baseline (Appendix Figure A1). These factors may contribute to a high degree of present bias and inability to fully internalize the distant health costs of tobacco consumption even when most *char*‐dwellers know of the associated negative health costs.

## 3 EXPERIMENTAL DESIGN AND EMPIRICAL STRATEGY

We designed two behavioral interventions to reduce tobacco consumption among the *char*‐dwellers in collaboration with MOMODa Foundation, a development research organization, and Samaj Kallyan Sangstha Foundation, a non‐profit development organization. We focused on the *chars* because of the high levels of poverty, precarious living conditions, exceptionally high tobacco consumption rates, and the absence of other tobacco cessation programs.

### 3.1 Design

The experiment was a part of the Bangladesh Chars Tobacco Assessment Project 2018 survey (Fakir et al., 2018). We began with a comprehensive list of *chars* and households residing in the *chars* of Gaibandha obtained from a census by our 2 local partner organizations in early 2018. *Chars* tend to be small in size with an average of 100‐120 households per *char*. The majority of these households within a *char* know each other and communicate frequently. Assigning different households from the same *char* to the control and the intervention groups would have resulted in significant spillovers. On the other hand, the poor communication and transportation infrastructure between *chars* make spillover across *chars* unlikely (Kabir, 2006; Paul & Islam, 2015). To minimize spillovers, we used a two‐stage clustered randomized controlled trial design.^10^

**FIGURE 1:**
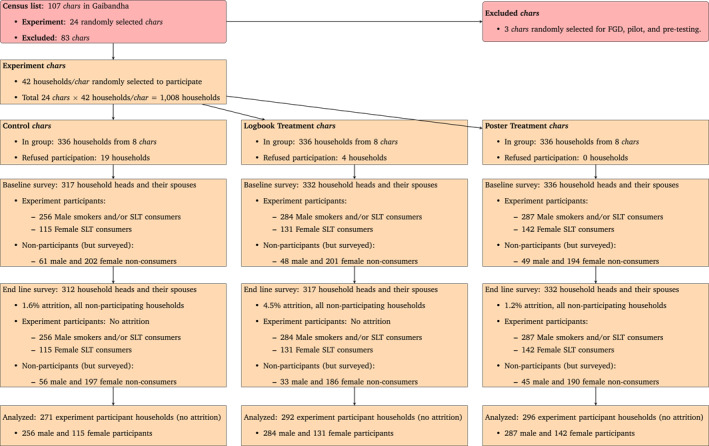
Description of the experiment sample. *Notes*: The baseline survey was conducted from November 10 to December 5, 2018. The intervention was implemented between December 10, 2018 and January 10, 2019. The end‐line survey was fielded from January 25 to February 10, 2019. Non‐participants households were surveyed but did not receive the intervention (logbook or poster) because they did not consume tobacco in any form. There was no attrition among participants. Only the sample of participants was used for the main analysis. There was no change in the tobacco consumption of non‐participant households

Based on the list constructed from the 2018 census, there were 107 *chars* in Gaibandha. Of these, we randomly selected 24 *chars* for the experiment. Eight *chars* each were randomly assigned to the control and the two intervention groups.^11^ Next, from the census list of all households residing in the 24 sampled *chars*, 42 households were randomly selected from each *char* for a targeted total of 1008 households. From each household, only the household head and their partner were interviewed. Therefore, we have information from one male and one female per household. Of them, none, one, or both might have consumed tobacco. A total of 23 households (2.3%) refused to participate in the surveys, 19 of them from the control group chars. Among the remaining 985 households, a total of 827 males and 388 females consumed tobacco in some form. They became the experiment participants. Note that all experiment participants and non‐participants are in the surveyed households, with 158 non‐participant males and 597 non‐participant females who did not consume tobacco.^12^ Thus, while the survey sample was selected from the entire *char* population, only households with household heads and/or spouses who consumed tobacco became the experiment participants. We conduct our main analyses using this sample of experiment participants. Please refer to Figure 1 for a detailed breakdown.

**FIGURE A2:**
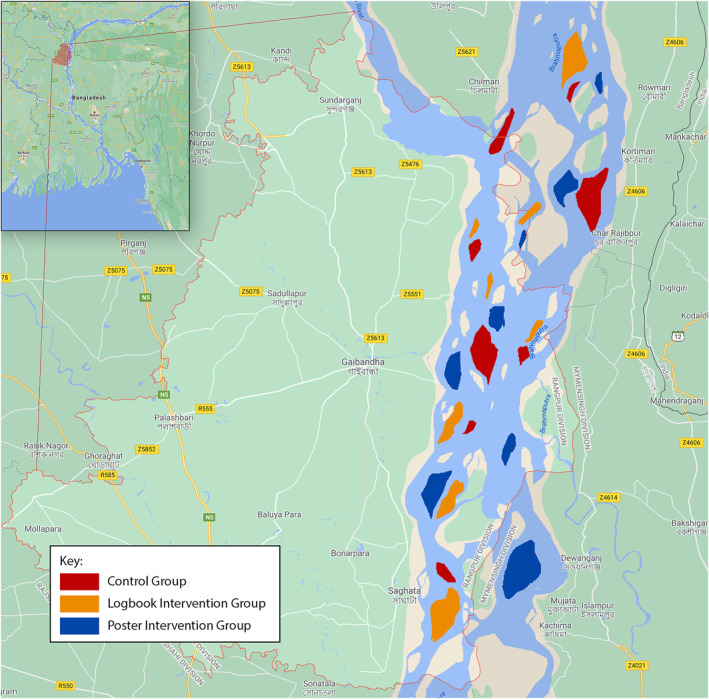
Distribution of experiment control & treatment *chars* in Gaibandha of northern Bangladesh

The final sample of participants was distributed as follows: (i) *No intervention* ‐ 256 male and 115 female participants from 271 experiment participant households in 8 *chars*; (ii) *LT* ‐ 284 male and 131 female participants from 292 experiment participant households in 8 *chars*; and (iii) *PT* ‐ 287 male and 142 female participants from 296 experiment participant households in 8 *chars*. The enumerators interviewed the household head and their spouse separately in the absence of their partner under a non‐disclosure clause explicitly mentioned to each participant. Appendix Figure A2 shows a map of the distribution of control and treatment *chars* of the study.

### 3.2 Interventions

**FIGURE A3:**
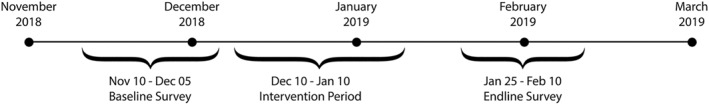
Project timeline

The baseline survey was done in November 2018, and the interventions ran for 4 weeks between December 2018 to January 2019. The end‐line survey followed 2 weeks after the end of the intervention period in February 2019 with no attrition among experiment participants.^13^ Appendix Figure A3 provides the timeline of the project.

**Group 01: No Intervention.** This reference (control) group did not receive any intervention. Each participant was interviewed in the baseline and the end line surveys, which were presented to them as part of a health checkup. All participants, including participants from the two treatment groups, were informed of their respiratory health status based on their forced expiratory volume (FEV1) measurements. FEV1 is a measure of the maximum amount of air an individual can forcefully exhale in 1 second. Persistent, heavy smoking leads to a rapid decline in FEV1 (Lange et al., 1989). FEV1 is used to determine the severity of a patient's lung damage and can help in diagnosing chronic obstructive pulmonary disease.

**Group 02: LT.** The LT required participating adult household heads and their spouse to record their total smoking and SLT consumption, and its estimated monetary cost, in a pictorial logbook daily. The idea here is that if participants observe their cumulative spending on tobacco regularly, the self‐actualization of the high monetary costs may motivate them to lower their tobacco consumption.

**FIGURE A4:**
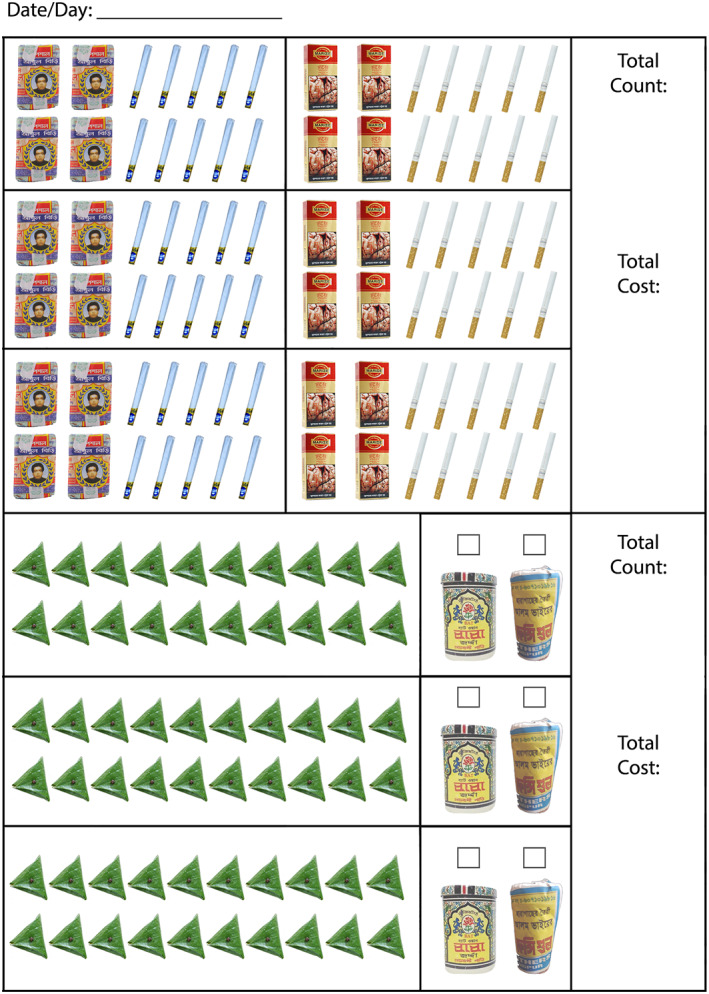
Logbook treatment: Sample logbook entry page *(translated)*

Due to high illiteracy among adults in the *chars* and to ensure that participants do not struggle in making their daily entries, we designed count‐based pictorial logbooks with feedback from focus group discussions and pre‐tests with participants of similar demography outside the experiment sample. Any text used in the logbook was in the local language, *Bangla*. Participants made entries based on tally counts for units of cigarettes, *bidis*, and/or SLT consumed and wrote down the estimated monetary value. We provided male and female participants with separate logbooks for individual entries (Appendix Figure A4). Development workers explained the record‐keeping procedure to the participants in detail and practiced three entry trials with each participant. To ensure compliance, the development workers took two steps. First, they made a phone call to the participants at a random time during the day once a week and reminded them to make their daily logbook entry. Second, they made one random in‐person visit during the 4‐week intervention period to check if the participants were up‐to‐date with their entries.^14^

Since we do not use the data entered into the logbooks, the accuracy of the entries does not matter. What is important is the repeated act of record‐keeping itself. Making frequent entries requires conscious acknowledgment of the quantity and frequency of tobacco intake. The tallying at the end of the day provides an estimate of the daily expenditure behind tobacco. This repeated self‐reminder is the nudge and the logbooks are simply the means to that end.

**FIGURE A5:**
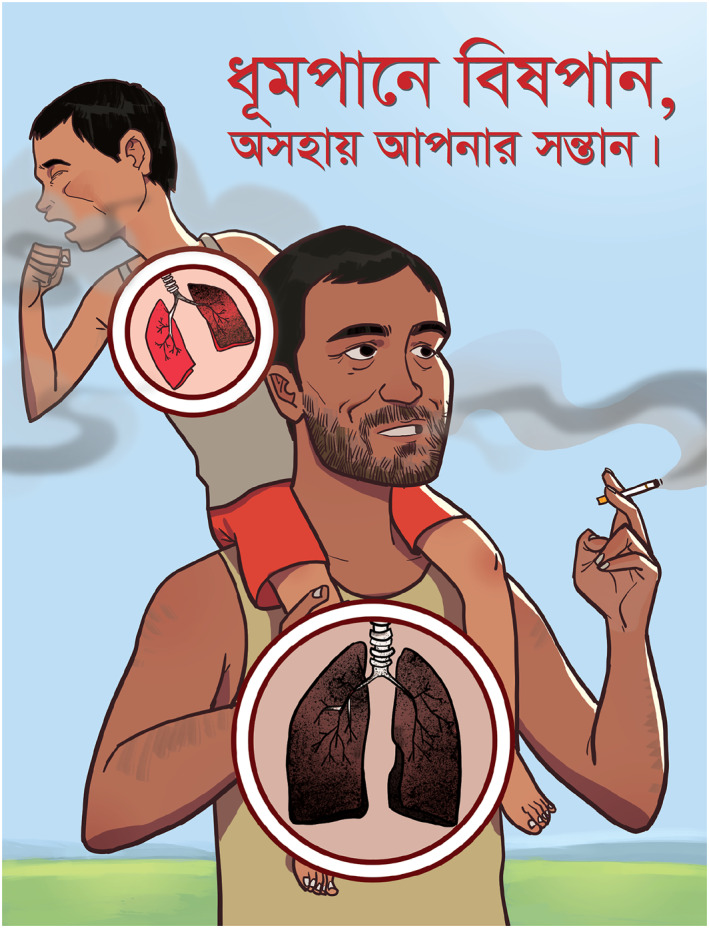
Poster treatment: Smoking Poster. **Top:** “Smoking is breathing poison,” **Bottom:** “that leaves your children helpless.”

**FIGURE A6:**
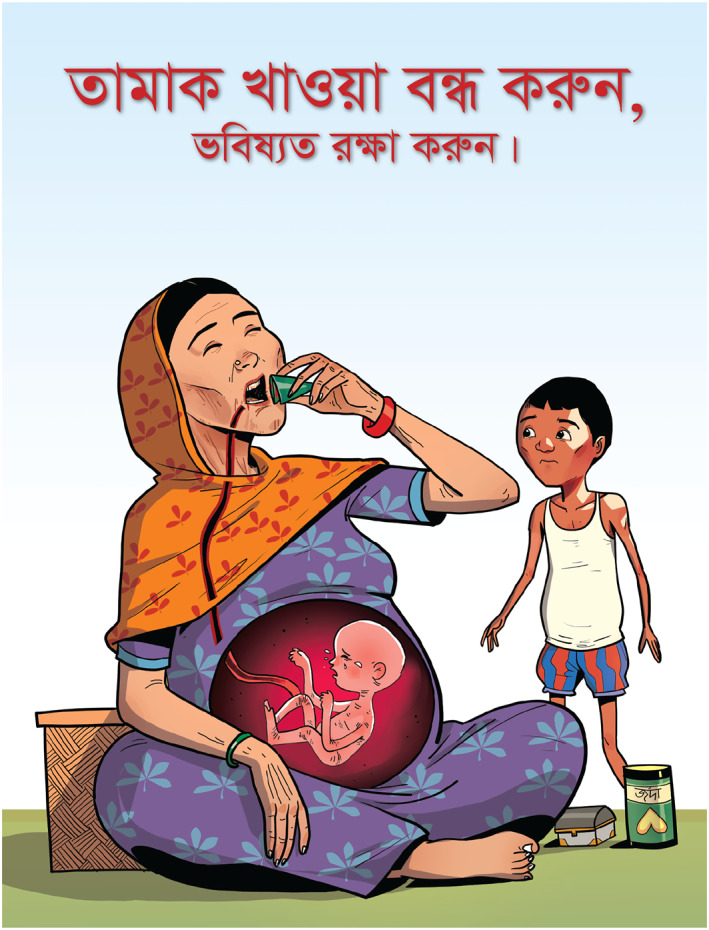
Poster treatment: smokeless tobacco (SLT) Poster. **Top:** “Stop tobacco intake,” **Bottom:** “protect your next generation.”

**Group 03: PT.** The PT placed two graphical posters in the sleeping quarters of the participating household head and the spouse. These posters were visual reminders of the health costs of smoking. Here, we wanted to investigate the extent to which the participants respond to reminders about the future health costs on themselves and their families (Kuehnle, 2019; Thrasher et al., 2011). One poster depicted a male giving a piggyback ride to a child while smoking a cigarette (Appendix Figure A5). The poster showed the harmful effects of smoking on the male, and that of second‐hand smoke on the child. The other poster showed the adverse effect of SLT consumption on a pregnant woman and her *in‐utero* child (Appendix Figure A6).

Warning labels on tobacco product packaging may be ineffective in Bangladesh because *bidis* and cigarettes are often bought as single sticks. At baseline, majority of male smokers made single‐stick purchases (40%–45% for *bidis* and 55%–60% for cigarettes). In addition, field observations revealed that packaging of *bidis* available in the *chars* did not abide by government regulations of warning labels covering at least 50% of tobacco packaging (NTCC, 2013; Rahman et al., 2019). Therefore, the experiment opted for a household‐level intervention. The artwork, the message, and the design of the posters were decided based on focus group discussions and in consultation with participants of similar demography but outside the experiment sample. This was to ensure that all participants, including those who are unable to read, can visually relate to the message conveyed in the posters. Any text used in the posters was in the local language, *Bangla*. The artwork of the posters is not as graphic as the typical warning labels on tobacco product packaging in Bangladesh. The decision was based on feedback from focus group discussions as many participants would not have agreed to hang such posters in their sleeping quarters. With the participants' consent, we hung the posters at a location such that they remain visually unobstructed and participants agree not to move them for the duration of the intervention. One random in‐person visit during the 4‐week intervention period was made to check the posters were still at the designated location.

At the end of the intervention period, the development workers visited the participants and collected the logbooks and the posters while also noting the participants' degree of compliance. Even though the participants were kept blind to the experiment's motive, respondents may feel a desire to under‐report subjective measures of tobacco intake at the end line. We conducted the end line survey with a 2‐week lag to allow some time for the participants to dissociate themselves from their respective intervention, hoping this would counteract this desirability bias to some extent. This is also the reason why the enumerators, who carried out the baseline and end line surveys, were different from the development workers who executed the interventions. Finally, measures of CO are not prone to such biases.

### 3.3 Data

Trained enumerators collected the vast majority of socio‐economic and self‐reported tobacco intake data from the household heads and their spouses, and experienced nurses took the CO and FEV1 measurements for better accuracy. The two main outcome variables we use in our empirical analysis are self‐reported tobacco intake and breath CO measurements.

*Self‐reported tobacco intake.* The baseline and end‐line survey collected information on tobacco consumption in the day prior to the interview. To facilitate better recall, we broke the day, from the time participants woke up to the time they went to bed, into three‐hour intervals.^15^ We then added the reported quantities in each three‐hour interval to arrive at the final measure of tobacco consumption during the day. For smoked tobacco, the total number of cigarette and/or *bidi* sticks were noted. This is available only for male participants as none of the females reported smoking. Since there is no discreet measure of SLT consumption (such as sticks for smoking tobacco), enumerators collected data on the frequency of SLT intake, including information on the type of SLT. The measures of both smoking tobacco and SLT included the consumption of any tobacco participants may have received from their employer (as is common for daily agriculture workers) or other acquaintances. The enumerators also noted the brands of the smoked and smokeless tobacco consumed.

We calculated the total expenditure on smoking tobacco by multiplying the number of sticks of cigarettes and/or *bidis* consumed with their brand‐specific prices. Total expenditure on SLT is similarly calculated using per “intake” prices of the consumed product, treating each intake as a unit. For example, if a participant reported that a 50 g container of *Shova zarda* costing Bangladeshi Taka (BDT) 20 lasts him or her for 4 days, with 10 daily intakes, it would mean that he or she consumed roughly 1.25 g per intake, costing BDT 0.5 per unit of *zarda* consumption. This approach takes into account that unlike smoking tobacco, where the per stick tobacco content is the same, different people may take varying quantities of SLT per intake. It provides a more standardized approximation for comparing SLT consumption expenditure values. Expenditure behind smoking tobacco and SLT of the partners is then summed to estimate total tobacco expenditure in a household.

*Breath carbon‐monoxide (CO) measurements.* Since breath CO measurements are sensitive to smoking tobacco (and not SLT) intake, we take CO measurements for male participants only. We use a Bedfont iCO Smokerlyzer machine to measure the CO levels. The Smokerlyzer is used widely in public health studies (Deveci et al., 2004; Muraven, 2010). The machine reports breath CO in 7 ordinal discrete categories of 0–6, 7–10, 11–15, 16–20, 21–25, 26–30, and 31+ parts per million (ppm). Nurses experienced in using the machine took the measurement between 4 and 6 PM in both baseline and end line surveys. The timing was important since the CO measurement is sensitive to smoking in the 8 h prior to the time of measurement. The nurses asked the participants to slowly exhale into the Smokerlyzer machine, fully emptying their lungs for as long as possible, at a steady pace after holding their breath for 15 s. Participants were permitted a trial and the second reading was recorded. Since ambient CO can affect the Smokerlyzer readings, measurements were taken in areas with no gas radiant heaters, open fires, or stoves (Cox & Whichelow, 1985). The CO readings provide an objective measure of smoking intensity free from self‐reporting or recall bias.

While we took precautionary steps to ensure self‐reported tobacco intake measures are accurate, the concern about under‐reporting to conform with the “socially desirable” outcome remains. Thankfully, the objective CO readings enable us to check if male participants under‐reported smoking tobacco consumption in the end line survey. For this, we regress *Sticks*_*it*_ on *CO*_*it*_, $CO_{it}^{2}$, and their interaction with a *Endline*_*t*_ dummy indicator and the *Treated*_*i*_ dummy. The interaction with the *Endline*_*t*_ dummy allows us to control for any secular trends in reporting that were unrelated to the experiment. An insignificant joint test of significance for *Endline*_*t*_ × *Treated*_*i*_ and its interactions with *CO*_*it*_ and $CO_{it}^{2}$ indicated that reporting did not change differently for treatment and control groups between the baseline and the end line.^16^ This improves confidence in the accuracy of the self‐reported survey measures.

The baseline survey also collected information on basic demographics, the number of children in the household, the participant's education, respiratory health status, time and risk preference, and experience of disruptive life events. Appendix Table A2 details the construction of variables and provides descriptive statistics. The nurses used a portable Microlife PF 100 Spirometry machine to measure participants' FEV1. We informed all participants about their measured respiratory health status. Hypothetical reward questions were used to elicit participant risk and time preferences. Risk preference was elicited following Fakir (2021) that prompted participants to choose between option *A*, a safe lottery of *w* for sure, and option *B*, a risky lottery of either 50 : 50 or 90 : 10 chance of *w* − *x* or *w* + *y* (Cox & Sadiraj, 2008; Harrison et al., 2017). Time preference was elicited using choices between smaller sooner rewards versus larger later rewards at varying discount rates to identify a participant's switching choice. We used two indicators of shocks faced by the households as proxies for stress. The first is a binary of any natural disaster(s) participants' faced in the 12 months prior to the survey, like flooding, land erosion, tropical storms, or livestock diseases, that affected them or their livelihood. The second is a count of the number of times the participant relocated *chars* within his or her lifetime and is a long‐term indicator of repeated exposure to stress.

### 3.4 Sample characteristics and balance

We begin by checking if the control and treated participants appear balanced among their observable traits at baseline. Table 1 provides the control group mean for each baseline characteristic or outcome, the coefficients and robust standard errors from a regression of each baseline characteristic or outcome on dummy variables indicating the logbook and the poster treatments, and *p‐values* from a test of equality of the two treatment dummy coefficients. We also report a Hotelling multivariate test of equality. Households, on average, consist of between four and five members, and roughly 35% of households have children below 5 years of age, with over 80% owning some land in the *chars*, inclusive of their homestead. Males are older in the sample with an average of 43 years while females average 36 years. Males are also less educated, have greater symptoms of lung disease, are mostly agriculture workers, were more exposed to anti‐tobacco campaigns in the past 12 months, and receive far greater tobacco from employers than females. Panel A provides balance checks of selected socio‐economic characteristics as a randomization check. Panel B further shows balance checks of outcome variables. Overall, baseline differences in means across control and intervention groups are statistically insignificant at conventional levels.

**TABLE 1:** Sample characteristics and baseline balance tests

	Control mean	*β*_*Poster*_	SE (*β*_*Poster*_)	*β*_*Logbook*_	SE (*β*_*Logbook*_)	*β*_*Poster*_ = *β*_*Logbook*_
	(1)	(2)	(3)	(4)	(5)	(6)
**Panel A: Demography & socio‐economic characteristics**						
Household						
Household size	4.435	0.181	(0.120)	0.088	(0.113)	0.573
Consumption quintile	5.306	0.178	(0.230)	0.062	(0.234)	0.724
Any land ownership	0.834	−0.001	(0.030)	0.041	(0.028)	0.306
Any child <5 years	0.352	0.025	(0.038)	0.020	(0.038)	0.926
Agriculture household	0.741	0.055	(0.039)	0.045	(0.035)	0.849
Shocks faced in past 12 months	0.605	0.016	(0.039)	0.027	(0.040)	0.844
Relocation ≤ 5	0.539	0.012	(0.038)	−0.038	(0.039)	0.359
*Hotelling p‐value*	‐	0.164	‐	0.209	‐	
Males						
Age (in years)	43.093	0.583	(1.012)	0.772	(1.019)	0.895
No education	0.645	−0.016	(0.039)	−0.030	(0.039)	0.800
Primary education	0.206	0.017	(0.032)	0.021	(0.033)	0.931
Higher education	0.149	−0.001	(0.030)	0.009	(0.030)	0.814
Lung damage	0.462	0.009	(0.039)	−0.021	(0.040)	0.591
Awareness campaign exposure	0.296	−0.016	(0.037)	−0.036	(0.037)	0.702
Employer provides tobacco	0.465	−0.022	(0.040)	0.022	(0.040)	0.437
*Hotelling p‐value*	‐	0.301	‐	0.125	‐	
Females						
Age (in years)	35.247	1.104	(0.843)	1.307	(0.841)	0.865
No education	0.539	0.009	(0.039)	−0.004	(0.039)	0.814
Primary education	0.224	0.020	(0.033)	0.025	(0.032)	0.913
Higher education	0.237	−0.029	(0.031)	−0.021	(0.031)	0.855
Lung damage	0.391	−0.007	(0.038)	0.037	(0.039)	0.419
Awareness campaign exposure	0.069	0.002	(0.020)	−0.021	(0.019)	0.405
Employer provides tobacco	0.019	−0.004	(0.010)	−0.013	(0.009)	0.504
*Hotelling p‐value*	‐	0.626	‐	0.175	‐	
**Panel B: Outcome variables**						
Household						
Breath CO level	4.478	0.113	(0.210)	0.171	(0.208)	0.844
Total tobacco expenditure	22.624	−0.333	(1.414)	−0.306	(1.309)	0.988
Smoking tobacco expenditure	14.407	−0.231	(0.962)	−0.870	(0.928)	0.633
Cigarette sticks	6.328	0.261	(0.405)	0.333	(0.406)	0.900
Bidi sticks	13.751	0.326	(1.027)	0.457	(1.045)	0.929
SLT expenditure	8.217	−0.102	(0.662)	0.564	(0.561)	0.443
SLT frequency	2.012	0.344	(0.256)	0.483	(0.299)	0.724
*Hotelling p‐value*	‐	0.152	‐	0.081*	‐	
Males						
SLT expenditure	1.988	0.058	(0.551)	0.214	(0.416)	0.821
SLT frequency	3.338	0.195	(0.233)	0.395	(0.247)	0.556
*Hotelling p‐value*	‐	0.233	‐	0.113	‐	
Females						
SLT expenditure	6.230	0.316	(1.139)	−0.228	(1.054)	0.955
SLT frequency	3.597	−0.064	(0.271)	0.136	(0.386)	0.672
*Hotelling p‐value*	‐	0.754	‐	0.138	‐	

*Note*: Column (1) provides the control mean at baseline. Columns (2) and (4) provide the coefficient from a regression of the variable on respective treatment dummy at baseline, while columns (3) and (5) provide the respective robust standard errors. Column (6) reports *p‐values* from a test of equality on the two treatment dummy coefficients. The *Hotelling* multivariate test *p‐value* for equality assesses whether respective treatment dummy is equal to 0 jointly across all (set of) baseline characteristics.

Abbreviations: CO, carbon‐monoxide; SLT, smokeless tobacco.

***p < 0.01, **p < 0.05, *p < 0.1.

### 3.5 Empirical strategy

McKenzie (2012) shows in experiments with a single baseline and one follow‐up survey, power is maximized when an end‐line outcome is regressed on the treatment measure conditional on the outcome's baseline value. Following the methodology, we estimate^17^:

(1) $$Y_{ihE}=\alpha +\beta_{1}T_{1ih}+\beta_{2}T_{2ih}+\beta_{3}Y_{ihB}+\varepsilon_{ih}$$

where *Y*_*ihE*_ is the outcome of interest at end line, *T*_1*ih*_ the LT dummy, *T*_2*ih*_ the PT dummy, *Y*_*ihB*_ the measure of the outcome at baseline, and *ɛ*_*ih*_ the random error. We cluster the standard errors at the *char* level. Our main outcomes, all collected by enumerators during the survey, are the usual daily expenditure on tobacco (smoked and smokeless), the number of sticks/frequencies of smoking/SLT consumed per day, and breath carbon‐monoxide (CO) measurements for smokers.^18^ For every outcome, we also test whether the estimated effects of the interventions differ from each other (*β*_1_ = *β*_2_).

Since there are several outcomes we examine, we also report adjusted FWER *p*‐values following Westfall and Young (1993). The method uses 1000 replications of bootstrapped samples to adjust for multiple hypotheses testing with correlated outcomes. Another concern is the low number of clusters (total of 24 *chars*). With small number of clusters, appropriate cluster‐robust standard errors can still lead to non‐trivial over‐rejection. To address this, we report *p*‐values from the wild bootstrap‐*t* clustering procedure that provide asymptotic refinement, hereafter Cameron‐Gelbach‐Miller (CGM) *p*‐values (Cameron et al., 2008; Djogbenou et al., 2019). As Cameron et al. (2008) shows, the CGM *p*‐values provide more reliable rejection rates even with as low as six clusters and no noticeable loss of power. Roodman et al. (2019)'s *boottest* command in Stata is used to compute the CGM *p*‐values. Another relatively common approach to circumvent the problem of faulty *t*‐ratios due to low number of clusters is utilizing the RI technique (Fisher, 1925; Rosenbaum, 2002; Splawa‐Neyman et al., 1990). The RI *p*‐values take into account sampling variability based on the set of possible clustered assignments. We also compute and report RI *p*‐values, using Hess (2017)'s *ritest* command in Stata, by randomly shuffling the treatment status 1000 times (Young, 2019).

## 4 RESULTS

### 4.1 Main results

Table 2 presents the main results.^19^ Both the LT and the PT caused a strong significant decrease in breath CO level and total tobacco expenditure (sum of tobacco use expenditure for the two partners). The LT caused around a 10% decrease in breath CO level $\left(\frac{-0.434}{4.304}\times 100\right)$ relative to the control group. Breath CO levels were measured for males only since none of the females smoked. It also caused a 22.8% decrease in household total expenditure on tobacco ([ exp(−0.259) − 1] × 100).^20^ The PT had a marginally smaller effect (−8.7%) on breath CO level and a larger effect (−25.3%) on household tobacco expenditure. However, the estimated effects of the two interventions are not statistically different. Adjusting the *p*‐values based on the FWER, the CGM, and the RI methods do not change the findings. The short‐lived interventions appear to have affected participants' choices, at least in the short run.^21^

**TABLE 2:** Impact on breath CO levels and household tobacco expenditure

	Breath CO level	Total tobacco expenditure (*log*)
	(1)	(2)
Logbook intervention (LT)	−0.434***	−0.259***
	(0.101)	(0.058)
Poster intervention (PT)	−0.375***	−0.292**
	(0.130)	(0.136)
FWER *p*‐value (LT)	0.023	0.000
FWER *p*‐value (PT)	0.045	0.000
CGM *p*‐value (LT)	0.011	0.000
CGM *p*‐value (PT)	0.019	0.000
RI *p*‐value (LT)	0.013	0.000
RI *p*‐value (PT)	0.014	0.000
LT = PT *p*‐value	0.720	0.823
Control mean of DDV	4.304	2.565
Observations	827	859

*Note*: Coefficients are intent‐to‐treat estimates. Robust standard errors clustered at the *char*‐level are in parentheses. All regressions control for the baseline value of their respective outcomes. “Total Tobacco Expenditure (log)” in column (2) measures the sum of tobacco use for the two partners while the “Breath CO Level” in column (1) is for males only (since none of the females smoked). Total tobacco expenditure are the log of winsorized (at the 99% level) daily values in BDT. CO levels are measured in 7 discrete categories of 0–6, 7–10, 11–15, 16–20, 21–25, 26–30, and 31+ parts per million (ppm). CGM *p*‐values are calculated using the wild bootstrap‐t clustering method (Cameron et al., 2008). RI *p*‐values are the Young's randomization inference based *p*‐values with 1000 replications (Young, 2019).

Abbreviations: CO, carbon‐monoxide; DV, dependent variable, FWER, family‐wise error rate; LT, Logbook intervention; PT, poster intervention; RI, randomization inference.

***p < 0.01, **p < 0.05, *p < 0.1.

Next, we attempt to understand potential pathways through which the interventions affected the outcomes in Table 2. We begin by examining the impact of the interventions on a set of self‐reported outcomes. Panel A of Table 3 reports the treatment effect on the perceptions around the health effects of tobacco consumption.^22^ First, as is clear from the control group mean of the dependent variables, an overwhelming majority of participants understood that tobacco consumption is harmful to their own and their children's health (further shown in Panel C of Appendix Figure A1). Next, the LT did not change people's perception of the harmful effects of tobacco consumption. This is expected because the LT did not provide any information about the health effects of tobacco consumption. In comparison, the PT increased the awareness about the harmful effects of SLT consumption by around 10%. Therefore, the poster treatment not only made the information about the health costs of smoking more *salient*, it also brought *new* information about the health costs of consuming SLT to some. It did not affect the perceptions around the effect of smoking. Panel B presents the effect on self‐reported smoking behavior. The control mean of the outcome variables show that the levels of smoking at schools, health facilities, and playgrounds were much lower than at home, or at *bazaars* (markets; further shown in Panel B of Appendix Figure A1). Only the PT affected self‐reported smoking behavior at different venues. It caused a decrease in smoking at home, where the posters were on display, and at playgrounds. It appears that men might have internalized the message from the posters.^23^ The effect of the two interventions on tobacco consumption must be interpreted against these findings.

**TABLE 3:** Impact on perceptions about tobacco consumption and smoking behavior

	Panel A: Perception about the effects of tobacco consumption				
	Second‐hand smoke is harmful for […]		SLT is harmful for […]		*Bidis* are more hamful than cigarettes
	Adults	Children	Adults	Children	
	(1)	(2)	(3)	(4)	(5)
Logbook intervention (LT)	0.020	0.001	0.003	−0.010	0.034
	(0.048)	(0.054)	(0.029)	(0.027)	(0.044)
Poster intervention (PT)	−0.036	−0.012	0.081***	0.068***	0.026
	(0.058)	(0.059)	(0.024)	(0.023)	(0.038)
LT=PT *p*‐value	0.457	0.871	0.038	0.028	0.891
Control mean of DV	0.741	0.762	0.828	0.799	0.627
Observations	827	827	827	827	827

	Panel B: Self‐reported smoking behavior				
	In the past week, did you smoke at […]?				
	Home, daily	Schools	*bazaars*	Health facilities	Playgrounds
Logbook intervention (LT)	0.056	0.005	0.022	−0.006	−0.010
	(0.074)	(0.025)	(0.073)	(0.034)	(0.034)
Poster intervention (PT)	−0.111**	0.020	0.065	0.039	−0.124**
	(0.045)	(0.026)	(0.091)	(0.059)	(0.054)
LT=PT *p*‐value	0.054	0.678	0.712	0.509	0.074
Control mean of DV	0.801	0.193	0.774	0.079	0.434
Observations	827	827	827	827	827

*Note*: Coefficients are intent‐to‐treat estimates. The sample consists of male participants only. Female participants were not enumerated on these questions. Robust standard errors clustered at the char‐level are in parentheses. All regressions control for the baseline value of their respective outcomes. All outcomes in Panels A and B are binary indicators that take value “1” if the respondent agree with the statement, “0” otherwise. In Panel B, “Smoked at home” takes value “1” if the participant responded with “daily” to the following question: “In the past week, how frequently did you smoke inside your home?”.

Abbreviations: DV, dependent variable; LT, Logbook intervention; PT, poster intervention; SLT, smokeless tobacco.

***p < 0.01, **p < 0.05, *p < 0.1.

In Table 4, we dig deeper into the estimated effects and examine the effect of the interventions on different measures of tobacco intake intensity separated by broad product categories. Both interventions decreased expenditure on smoking by males.^24^ As column (2) suggests a decrease in cigarette consumption was one of the driving factors. Interestingly, the effect of the interventions on the consumption of cheaper tobacco products differed significantly. While the PT had a significant negative effect on *bidi* consumption, the LT did not. The LT led to a significant increase in the sum of the two partners' expenditure on SLT and on their frequency of SLT consumption. In comparison, the PT had a marginal negative effect on SLT consumption.

**TABLE 4:** Impact on the type of tobacco consumed

	Smoking tobacco	Cigarette sticks	Bidi sticks	Smokeless tobacco	Smokeless frequency
	Exp. (*log*)			Exp. (*log*)	
	(1)	(2)	(3)	(4)	(5)
Logbook treatment (LT)	−0.281***	−2.282***	−0.561	0.171**	0.528**
	(0.043)	(0.450)	(0.527)	(0.059)	(0.213)
Poster treatment (PT)	−0.237***	−1.729***	−2.331*	−0.125*	−0.394
	(0.051)	(0.243)	(1.237)	(0.073)	(0.247)
FWER *p*‐value (LT)	0.000	0.000	0.365	0.055	0.022
FWER *p*‐value (PT)	0.000	0.000	0.033	0.098	0.111
CGM *p*‐value (LT)	0.000	0.000	0.228	0.027	0.009
CGM *p*‐value (PT)	0.000	0.000	0.048	0.155	0.164
RI *p*‐value (LT)	0.000	0.000	0.263	0.033	0.015
RI *p*‐value (PT)	0.000	0.000	0.067	0.187	0.182
LT=PT *p*‐value	0.510	0.280	0.188	0.002	0.005
Control mean of DV	2.110	5.142	13.363	1.644	2.490
Observations	827	827	827	859	859

*Note*: Coefficients are intent‐to‐treat estimates. Robust standard errors clustered at the *char*‐level are in parentheses. All regressions control for the baseline value of their respective outcomes. Since only males smoked, “Smoking Tobacco Exp. *(log)*,” “Cigarette Sticks,” and “Bidi Sticks” in columns (1), (2), and (3) reflect expenditure behind smoking tobacco by males only. “Smokeless Tobacco Exp. *(log)*” and “Smokeless Frequency” in columns (4) and (5) is the sum for the two partners. Expenditure values in columns (1) and (4) are the log of winsorized (at the 99% level) daily values in BDT. Stick counts in columns (2) and (3) and frequency in column (5) are respective daily intake. CGM *p*‐values are calculated using the wild bootstrap‐t clustering method (Cameron et al., 2008). RI *p*‐values are the Young's randomization inference based *p*‐values with 1000 replications (Young, 2019).

Abbreviations: DV, dependent variable; FWER, family‐wise error rate; LT, Logbook intervention; PT, poster intervention.

***p < 0.01, **p < 0.05, *p < 0.1.

The differences are not surprising. The LT focused on the current costs of tobacco consumption, to which participants responded with measures to decrease their expenditure on tobacco.^25^ They switched to consuming more SLT, the cheaper tobacco products available in the *chars*.^26^ Since breath CO levels do not respond to SLT intake, we have no way of understanding if the LT reduced their overall tobacco intake. All we can deduce is that constant reminders about tobacco expenses had the expected effect —households reduced their tobacco expenditure by switching from cigarettes to SLT products. This is still problematic since the consumption of SLT products is also extremely detrimental to health. (Hoffmann & Djordjevic, 1997; Roosaar et al., 2008).

The PT reminded people of the distant costs tobacco consumption imposes on their own and their children's health. As discussed above, the harmful effects of SLT were also new information for some participants. Consistent with this, we find the reduction in cigarette consumption was not compensated by an increase in SLT consumption. There was a marginally significant decrease in expenditure on SLT as well. As pointed out in Table 3, the interventions did not change the perceptions about the harmful effects of cigarettes. Therefore, the reduction in cigarette consumption appears to result from the constant reminders alone. In comparison, the marginal reduction in SLT consumption is a consequence of both providing new health information and increasing the salience of the information with constant reminders.

Implementing the logbook and poster treatments cost around BDT 1100 (USD 13) and BDT 950 (USD 11) per participant, respectively.^27^ This is based on a back‐of‐the‐envelope calculation with an approximate cost of designing and printing information materials, daily wage rates of the development workers and nurses, transportation, allowances, and monitoring of treatment compliance for a 1‐month duration (excluding data collection/research costs). The logbook and poster interventions reduced daily household total tobacco expenditure by 22.8% (or BDT 5.16) and 25.3% (or BDT 5.72), respectively. Nargis et al. (2014) estimated a 4.9% reduction in daily cigarette consumption expenditure from a 10% increase in the price of cigarettes in Bangladesh. If we assume the authors' estimates to be comparable for all forms of tobacco, the logbook and poster interventions had impacts similar to 46.5% and 51.6% increases in the price of tobacco.^28^ Although larger in their impact, our interventions, like an increase in tobacco taxes, do not generate tax revenues and it is unclear if the effect of these interventions would hold up in the longer run. For these reasons, it is difficult to compare the cost‐effectiveness of the interventions. We could not find other tobacco cessation interventions in Bangladesh that also reported associated interventions costs.^29^

These findings have important policy implications. Unlike developed countries, where SLT products are less common, expenditure reminders in the presence of multi‐tiered tax instruments can backfire in developing countries, as people may switch to cheaper but harmful alternatives. Our results highlight the need for carefully designing these interventions, grounding them firmly in theory, so that such perverse substitution effects can be predicted and corrected. The findings from the PT point out that the salience of information about the distant health cost of tobacco consumption might be enough to discourage people from tobacco consumption if it is in the form of constant reminders that leverage their concern for their children.

### 4.2 Treatment effects by gender

To improve the design of such behavioral interventions, it is of value to identify participant groups that are most persuadable with different interventions. We attempt this by examining the heterogeneity in the impact of the two interventions. First, we investigate if the effectiveness of the interventions differed by gender. Recall that only the male participants smoked, but both males and females consumed SLT. Therefore, we can examine the heterogeneity by gender only for SLT consumption. We report the findings in Table 5. The adverse substitution towards SLT products because of the LT happened only for men. This is entirely expected since only men smoked cigarettes. The LT was associated with a decrease in SLT consumption for women, albeit with no statistical significance.^30^ The PT had a negative but insignificant effect on the SLT consumption of males and a significant negative effect on the SLT consumption of females. There are two potential reasons for this. First, females consumed SLT more than twice as often as males. It is likely that the posters had a bigger impact on participants who are more frequent users of SLT. Second, the poster warning the participants about the harmful effects of SLT depicted a pregnant woman consuming SLT. It is possible that for this reason women participants were affected more by these posters than male participants. If so, such nuances need to be taken into account when designing campaigns aimed at promoting health behavior.

**TABLE 5:** Impact on Smokeless (SLT) consumption by gender

	Males		Females	
	Smokeless tobacco products			
	Exp. (*log*)	Frequency	Exp. (*log*)	Frequency
	(1)	(2)	(3)	(4)
Logbook intervention (LT)	0.201**	0.670**	−0.083	−0.188
	(0.079)	(0.265)	(0.055)	(0.117)
Poster intervention (PT)	−0.036	−0.172	−0.113*	−0.246*
	(0.055)	(0.209)	(0.060)	(0.136)
FWER *p*‐value (LT)	0.021	0.022	0.373	0.319
FWER *p*‐value (PT)	0.544	0.538	0.080	0.082
CGM *p*‐value (LT)	0.015	0.016	0.347	0.302
CGM *p*‐value (PT)	0.547	0.527	0.071	0.077
RI *p*‐value (LT)	0.020	0.020	0.392	0.355
RI *p*‐value (PT)	0.572	0.546	0.076	0.072
LT=PT *p*‐value	0.014	0.013	0.713	0.747
Control mean of DV	0.911	1.528	1.358	3.455
Observations	827	827	388	388

*Notes*: Coefficients are intent‐to‐treat estimates. Robust standard errors clustered at the *char*‐level are in parentheses. All regressions control for the baseline value of their respective outcomes. Expenditure values in columns (1) and (3) are the log of winsorized (at the 99% level) daily values in BDT. Frequency in columns (2) and (4) are respective daily intake. CGM *p*‐values are calculated using the wild bootstrap‐t clustering method (Cameron et al., 2008). RI *p*‐values are the Young's randomization inference based *p*‐values with 1000 replications (Young, 2019).

Abbreviations: DV, dependent variable; FWER, family‐wise error rate; LT, Logbook intervention; PT, poster intervention; SLT, smokeless tobacco.

***p < 0.01, **p < 0.05, *p < 0.1.

Next, we examine the heterogeneous effect of the interventions by whether the spouse also consumed tobacco. If the interventions affect participants from the same household independently, these interventions may have a higher marginal benefit in households where multiple members consume tobacco. It is also possible that multiple users in the same household enable each other's addiction and dampen the effect of the interventions. The findings in Table 6 indicate that it might be the latter. Males with a non‐consumer wife had a higher reduction in breath CO levels and expenditure on smoking compared to males with a tobacco‐consuming wife. Consistent with earlier results, these males in the LT group also switched to SLT more often. We do not find statistically different effects for females with husbands who did and did not consume tobacco. While the interaction coefficients for females are in the right direction, the lack of significance is probably a consequence of the fact that there are few households where the wife consumes tobacco and the husband does not.

**TABLE 6:** Impact on breath CO level by spousal tobacco intake

	Males				Females	
	Breath CO level	Smoking exp. (*log*)	SLT exp. (*log*)	SLT frequency	SLT exp. (*log*)	SLT frequency
	(1)	(2)	(3)	(4)	(5)	(6)
Logbook intervention (LT)	−0.276^++^	−0.213^+++^	0.102^++^	0.313^+^	−0.058	−0.129
	(0.121)	(0.059)	(0.041)	(0.179)	(0.142)	(0.115)
Poster intervention (PT)	−0.256^+++^	−0.201^+++^	−0.119	−0.062	−0.108	−0.208
	(0.057)	(0.067)	(0.074)	(0.117)	(0.062)	(0.277)
Spouse does not consume tobacco	−0.276^+^	−0.313^++^	−0.337^+++^	−0.544^+++^	−0.228^+^	−0.298^++^
	(0.132)	(0.132)	(0.122)	(0.125)	(0.124)	(0.119)
LT × spouse does not consume tobacco	−0.334^+^	−0.129^+^	0.115	0.408	−0.050	−0.012
	(0.178)	(0.067)	(0.090)	(0.299)	(0.180)	(0.209)
PT × spouse does not consume tobacco	−0.326^++^	−0.119^++^	−0.039	−0.258	−0.170	−0.195
	(0.127)	(0.052)	(0.064)	(0.175)	(0.102)	(0.154)

Overall effects on indicated category						
Logbook intervention (LT)	−0.610***	−0.342***	0.252**	0.721**	−0.108	−0.141
	(0.228)	(0.109)	(0.120)	(0.355)	(0.139)	(0.188)
Poster intervention (PT)	−0.582***	−0.321***	−0.158	−0.320	−0.278*	−0.403*
	(0.207)	(0.114)	(0.109)	(0.283)	(0.150)	(0.218)
LT=PT *p*‐value	0.881	0.893	0.009	0.080	0.747	0.792
LT=PT interaction *p*‐value	0.971	0.906	0.163	0.055	0.562	0.481
Control mean of DV	4.304	2.110	0.911	1.524	1.358	3.455
Observations	827	827	827	827	388	388

*Note*: Family‐wise error rate (FWER) *p‐values*: ^+++^ *p* < 0.01, ^++^ *p* < 0.05, ^+^ *p* < 0.1. Robust standard error *p‐values*: ***p < 0.01, **p < 0.05, *p < 0.1. Coefficients are intent‐to‐treat estimates. Robust standard errors clustered at the *char*‐level are in parentheses. All regressions control for the baseline value of the outcome. CO levels in column (1) are in 7 discrete categories of 0–6, 7–10, 11–15, 16–20, 21–25, 26–30, and 31+ parts per million (ppm); expenditure values in columns (2), (3), and (5) are the log of winsorized (at the 99% level) daily values in BDT; frequency values in columns (4) and (6) are counts of daily intake.

Abbreviations: DV, dependent variable; LT, Logbook intervention; PT, poster intervention; SLT, smokeless tobacco.

### 4.3 Exploratory heterogeneity analysis

In Table 7, we examine the heterogeneity in the impact of the two interventions on smoking by participant characteristics at baseline. Since we did not include this in the pre‐analysis plan, the following analyses should be considered exploratory (Olken, 2015).^31^ Appendix Table A2 defines the indicator variables we use for the heterogeneity analysis, along with the rationale for selecting the variable and the basis for respective cut‐offs.^32^ While the interaction effects have sizable magnitudes, they are not all significant. This could result from the small number of clusters we are working with. To be conservative, we discuss only those characteristics that are statistically significant as per the adjusted FWER *p‐values*.

**TABLE 7:** Heterogeneity in the impact on breath CO level (Male participants)

	Breath CO level								
	(1)	(2)	(3)	(4)	(5)	(6)	(7)	(8)	(9)
Logbook intervention (LT)	−0.388^++^	−0.229^++^	−0.278^++^	−0.323^+++^	−0.163^++^	−0.263^++^	−0.362^++^	−0.324^+^	−0.192^+^
	(0.163)	(0.097)	(0.117)	(0.108)	(0.077)	(0.101)	(0.155)	(0.148)	(0.087)
Poster intervention (PT)	−0.266^+^	−0.380^++^	−0.317^++^	−0.249^++^	−0.155^++^	−0.190^+^	−0.292^++^	−0.197^+++^	−0.152^+^
	(0.134)	(0.159)	(0.126)	(0.112)	(0.073)	(0.098)	(0.107)	(0.068)	(0.070)
LT × Lung damage	−0.329								−0.531
	(0.551)								(0.386)
PT × Lung damage	−0.298								−0.257
	(0.388)								(0.293)
LT × Risk averse		−0.205^+^							−0.439^+^
		(0.111)							(0.229)
PT × Risk averse		0.110							0.297
		(0.521)							(0.387)
LT × No shock			−0.539						−0.372
			(0.487)						(0.507)
PT × No shock			−0.589						−0.337
			(0.526)						(0.656)
LT × Relocation ≤5				−0.477^+^					−0.653^+^
				(0.241)					(0.317)
PT × Relocation ≤5				−0.417					−0.700
				(0.606)					(0.576)
LT × Tobacco exp >5.5%					−0.208^+^				−0.191
					(0.108)				(0.114)
PT × Tobacco exp >5.5%					−0.323				−0.211
					(0.481)				(0.395)
LT × Child <5 years						−0.273			0.093
						(0.584)			(0.388)
PT × Child <5 years						−0.516^+++^			−0.649^+++^
						(0.131)			(0.226)
LT × Any schooling							−0.116		−0.235
							(0.489)		(0.435)
PT × Any schooling							−0.403^+^		−0.546^++^
							(0.225)		(0.219)
LT × Patience (time pref.)								−0.130	0.039
								(0.329)	(0.420)
PT × Patience (time pref.)								−0.524^+^	−0.416
								(0.244)	(0.281)
**Overall effect on indicated category**									
Logbook intervention (LT)	−0.717	−0.434**	−0.817	−0.800**	−0.371*	−0.536	−0.479	−0.454	‐
	(0.423)	(0.172)	(0.755)	(0.304)	(0.201)	(0.630)	(0.549)	(0.369)	‐
Poster intervention (PT)	−0.564	−0.270	−0.906	−0.665*	−0.478	−0.706***	−0.695**	−0.720**	‐
	(0.421)	(0.395)	(0.528)	(0.368)	(0.449)	(0.219)	(0.302)	(0.362)	‐
LT = PT *p*‐value	0.563	0.418	0.821	0.634	0.940	0.604	0.710	0.436	0.720
LT = PT interaction *p*‐value	0.963	0.554	0.944	0.927	0.816	0.685	0.594	0.358	‐
Control mean of DV	4.304	4.304	4.304	4.304	4.304	4.304	4.304	4.304	4.304
Observations	827	827	827	827	827	827	827	827	827

*Note*: Family‐wise error rate (FWER) *p‐values*: ^+++^ *p* < 0.01, ^++^ *p* < 0.05, ^+^ *p* < 0.1. Robust standard error *p‐values*: ***p < 0.01, **p < 0.05, *p < 0.1. Coefficients are intent‐to‐treat estimates from fully saturated regressions. Robust standard errors clustered at the *char*‐level are in parentheses. All regressions control for the baseline value of their respective outcomes. CO levels are measured in 7 discrete categories of 0–6, 7–10, 11–15, 16–20, 21–25, 26–30, and 31+ parts per million (ppm). The construction of indicator variables is explained in Table A2.

Abbreviations: CO, carbon‐monoxide; DV, dependent variable; LT, Logbook intervention; PT, poster intervention.

The LT had a larger effect on relatively risk‐averse male participants. This is consistent with earlier studies that find risk aversion is negatively associated with smoking and relapse among smokers trying to quit (Anderson & Mellor, 2008; Goto et al., 2009). Interestingly, the PT did not have a large effect on risk‐averse participants. The LT also had a larger effect on those who had fewer than six *char* relocations during their lifetime. It is likely that frequent relocations force people to rely more heavily on smoking to cope with repeated disruptions, and those heavily reliant on smoking find it difficult to reduce their consumption (Becker & Murphy, 1988). Participants who spent over 5.5% (sample median) of their household budget on tobacco also responded more to the LT. This is perhaps because the LT was more revealing to them.

The PT had a larger effect on male participants with children under five. This is not surprising since the poster depicted the harmful effects of smoking on themselves as well as their children. For participants with children under five, the poster treatment is likely to have carried a heavier influence. The end‐line survey asked male participants about their emotional reactions to the posters. Participants, especially those with children under five, reported feeling sad, ashamed and disgusted by their habit of tobacco consumption. Appendix Table A8 reports the associations. The findings suggest that the PT worked by channeling the participants' concern for their children. Participants with any schooling and more patience also responded more to the poster treatment. Those with schooling might have better internalized the message in the poster. They might also be more patient (Jung et al., 2021). Posited by Becker and Murphy (1988) and verified in numerous studies, those who discount the future less are less likely to engage in harmful addictions (see Cawley and Ruhm (2011) for a review). In Appendix Table A9, we present the corresponding heterogeneity analysis with the male expenditure on tobacco as the dependent variable. The estimated associations are, in their directions and statistical significance, quite similar to those in Table A3, A4, A5, A6, A7, A8, A9.^33^

Together, the findings from the heterogeneity analyses point out that different approaches may be required to persuade different populations to reduce tobacco consumption (or in promoting other health behaviors). For patient and educated people with children, helping them acknowledge the distant negative health effects of smoking or other risky health behavior on themselves and their children might be enough. For those constantly exposed to disruptive life events, cost reminders might work better if substitution to inferior products is prevented. Participants with spouses who do not engage in risky behavior might be easier to dissuade. Careful consideration of such details to tailor behavioral interventions to the socio‐demographic characteristics of the target population of different regions can greatly increase the effectiveness of such interventions. While this can be more expensive than rolling out a singular intervention, regional targeting could be more cost‐effective if the gains are significant.

## 5 LIMITATIONS

The first limitation of the study is its statistical power. Although we find significant effects of the two interventions and conduct various robustness checks, more clusters and more observations from each cluster would have definitely helped to boost the power. It is possible that some of the estimated effects are statistically insignificant because of the low number of clusters.^34^ The brief intervention period implies we can make inferences only about the short‐term effects of the interventions. As Appendix Figure A3 shows, the gap between baseline and end line data collection periods was 6 weeks. The results may thus be driven by an initial adaptation effect that may not translate to long‐term behavior changes.^35^ A longer follow‐up would help to answer some of these questions.

The control group did not receive a placebo intervention. It is possible that a part of the effect we estimate is a consequence of being chosen to receive an intervention than the impact of the intervention itself. While the difference in the estimated effect of the two interventions makes it unlikely, we cannot completely rule it out. Unfortunately, due to limited resources, we had to proceed without a placebo. A design limitation was that we did not have a treated group that received both interventions. It would have been interesting to examine if the posters would have counteracted some of the substitutions toward SLT that occurred under the LT, and whether the two interventions would reinforce each other to produce an even larger impact. Future research studies should try to examine such complementarities. Another weakness arising from data limitations is that we cannot examine any other positive substitution effects, like increased expenditure on clothing or education, that might have resulted from the savings the households made.

This experiment was specifically designed as an anti‐tobacco campaign for the rural poor in the *char* regions. The unique demography and contextual setting of the *chars* may lessen the external validity of the findings. Replications outside *char* regions where tobacco penetration is not as high or where people are more educated may yield different results. Finally, it is difficult to comment on whether the interventions we examine are any easier to implement than taxes on tobacco, especially when they need to be scaled up to the national level. While implementing these interventions at a large scale might be easier in countries like Bangladesh where grassroots‐level involvement of NGOs is high, further research is necessary to verify this.^36^

## 6 CONCLUSION

From calorie labeling to emoticons on power bills, policies are increasingly using nudges to promote health behavior. But many such interventions are often without reasonable evidence of their effectiveness, proper understanding of the mechanisms, and assessment of possible unintended consequences. In an attempt to identify cost‐effective ways to reduce tobacco intake among a remote rural‐poor population in Bangladesh, we highlight such shortcomings. We implement two nudges. The first asked participants to maintain a logbook of their expenditure on tobacco to remind them of the monetary cost of their tobacco consumption. The second placed posters with graphical warnings about the harmful health effects of tobacco consumption on the users and their children.

We learn that the effect of nudges that stress the monetary cost, similar to price instruments like higher taxes, depends on the range and affordability of substitutes available on the market. In this case, cheaper alternatives to cigarettes were available in the form of SLT products. So, the increased salience of financial costs caused a substitution for cheaper tobacco products. An important takeaway is that policies that aim to motivate cessation by increasing the salience of expenditures should also point out the harmful effects of cheaper substitutes. Visual reminders, with information about the harmful effects of tobacco consumption on the participants and their children, were more effective in reducing smoking and SLT intake without any such substitution.

Male participants with spouses who did not consume tobacco were more likely to be affected by the interventions. Exploratory analyses show that the LT had a larger impact on male participants with greater risk‐averse attitudes, who had a lower number of lifetime *char* relocations, and those who spent higher proportions of total household expenditure on tobacco. Patient, educated male participants with children less than 5 years of age were more likely to respond to the PT. The heterogeneity in the estimated effects suggests that regional targeting of interventions based on the prevailing socio‐economic characteristics of the target population may be most suitable.

The findings also provide a cautionary tale to policymakers to be vigilant of unintended consequences when designing behavioral interventions. While behavioral economics provides powerful insights, applying them to the design of better policies is a challenge that requires adequate testing before wider execution. We must reemphasize that the findings discussed in this study explore the short‐term effects of these interventions. Future research must examine the longer‐term effects of such interventions to better understand how to reduce tobacco use in the long term. It is also important to explore the role of spillovers and social networks in promoting information salience for health decision making, something we could not do due to data limitations. Such spillovers, if present, could increase the cost‐effectiveness of these interventions. The findings from future research should be used to calibrate intervention design and improve targeting practices. Overall, our findings re‐emphasize the importance of evidence‐based policy design.

## CONFLICT OF INTEREST

None.

## Figures and Tables

**FIGURE A7:**
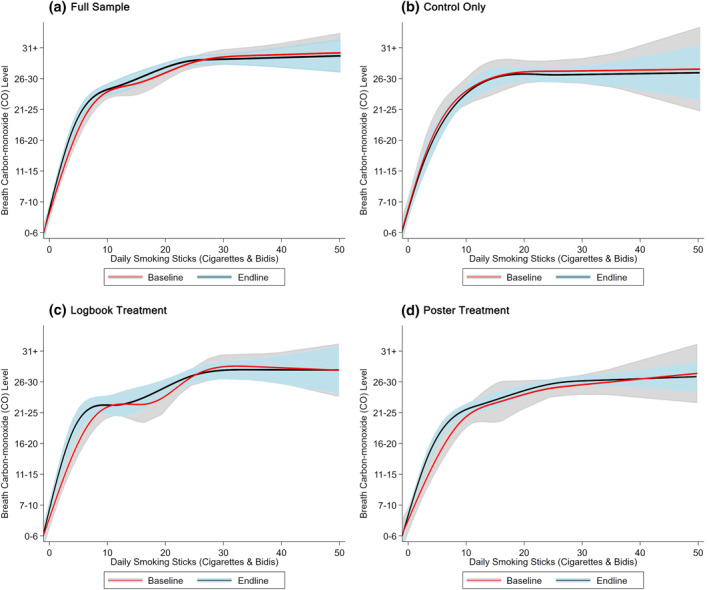
The relationship between carbon‐monoxide (CO) readings and reported total sticks count at baseline and end line. *Notes*: Semi‐parametric estimates, with 95% confidence interval, of the relationship between breath CO levels and reported total sticks count of cigarettes and/or *bidis* at baseline and end line

## Data Availability

Data subject to third party restrictions.

## ENDNOTES

^1^
Early criticism came from those opposed to “paternalistic” government intervention, arguing that such policies went against consumer sovereignty and freedom of choice and expression (Tollison & Wagner, 2012; Viscusi et al., 1992). Others have questioned the fairness of such taxes. They opined the tax burden may fall disproportionately on low‐income people who may be more likely to partake in the unhealthy behavior the policies are trying to discourage.

^2^
Some examples are the endorsement and adoption of behavioral economics by former US president Barack Obama and present US president Joe Biden and the establishment of the Behavioral Insights Team, or the “Nudge Unit”, by David Cameron's government in the United Kingdom in 2010 (Biden, 2021; Obama, 2015; Sunstein, 2021; Wintour, 2010).

^3^

*Bidis* are hand‐rolled, filter‐less smoking tobacco made from wrapping tobacco flake. A traditional method of tobacco use in South Asia, they are cheaper than regular cigarettes and are mostly consumed by the poor.

^4^
Since SLT is smokeless, the CO measurements cannot pick this substitution.

^5^
There are over 37 million tobacco consumers in Bangladesh. The Global Adult Tobacco Survey (GATS) suggests that the rural to the urban ratio of tobacco consumption increased from 1.18 to 1.24 between 2009 and 2017, with about 21 million tobacco consumers in rural Bangladesh (WHO, 2018a, 2018b).

^6^
While there is evidence to suggest that many behavioral interventions backfire, they are seldom reported in academic journals (Bolton et al., 2020; Stibe & Cugelman, 2016).

^7^
Perhaps, the closest studies are Baird et al. (2014), Paula et al. (2014), and Gong (2015) that document information provision about a person's HIV status can sometimes increase risky sexual behavior in HIV‐positive individuals in sub‐Saharan Africa. There is a dearth in the number of studies that investigate the effectiveness of information interventions in health. Previous works crowd in the area of education, such as Banerjee et al. (2010) in India, Pradhan et al. (2014) in Indonesia, and Lieberman et al. (2014) in Kenya. Olken (2007) study the efficacy of information in reducing corruption in village road project funds. Banerjee et al. (2018) evaluate the role of information in the delivery of poverty programs.

^8^
Appendix Table A1 provides an overview of the type of available tobacco products and their prices.

^9^

*Zarda* and *gul* are locally produced smokeless forms of tobacco. *Zarda* is normally chewed with betel leaf and areca nut, while *gul* is applied to the teeth and gums.

^10^
It is worthwhile to note that our outcomes are not statistically significantly different between baseline and end line in the control group, providing some assurance that there are no spillovers from the treatment *chars* to the control *chars*.

^11^
The two‐stage CRCT with a nested design comes at the cost of a relatively low‐powered experiment. As we discuss in Section 3.5, we use ANCOVA to boost power.

^12^
The non‐participants were also surveyed to examine if the interventions had any negative spillovers on those who did not smoke at baseline. End‐line interviews confirmed no negative spillovers with no new tobacco consumers.

^13^
There was no attrition from the experiment sample. Households heads and their spouses from all households in the experiment sample where at least one of them consumed tobacco at the baseline were interviewed again at the end line. There was some attrition of non‐participating surveyed households (those who did not consume tobacco). A total of 24 non‐participating surveyed households (2.4%) were lost between the 2 waves. Of these, 5 households (1.6%) were from the control group, 15 households (4.5%) were from the logbook treatment group, and 4 households (1.2%) were from the poster treatment group. The difference between the rate of attrition from the logbook treatment group and the control group was statistically significant (*p‐value* = 0.084). The difference between the poster treatment group and the control group was not significant (*p‐value* = 0.793). This is unlikely to be a problem because we do not use any information from non‐participating households for our main analyses.

^14^
It is perhaps worth mentioning that the development workers are locals who have a good rapport with and are respected by the participants. This played a key role in ensuring compliance. In the logbook treatment, about 77% of all participants made daily entries, while the remaining 23% (*n* = 95) had made entries weekly, with no statistically significant difference in weekly entries by groups. Segregated by gender, 24% of males (*n* = 68) and 21% of females (*n* = 27) had made weekly entries. An association check reveals no significant difference by compliance variation on outcome measures, reported in Panel A of Appendix Table A6. Further, while those with higher education had more compliance and conducted more regular daily logbook entries among both male and female participants, there is no statistically significant difference.

^15^
Smoking behavior may differ on workdays and rest days. The survey was planned such that tobacco consumption was collected for workdays. The data do not reflect tobacco consumption during rest days. The baseline and end‐line surveys were within 2 months of each other, both in the same season, to net out any seasonal trends in tobacco consumption. The surveys were fielded during the winter season when the *Rabi* crops, sown in winter and harvested in spring, were being sown into the fields. This also ensured that the workload remained fairly similar at baseline and end line.

^16^
We run the following double difference estimation: $Sticks_{it}=\alpha +\beta_{1}CO_{it}+\beta_{2}CO_{it}^{2}+\beta_{3}Endline_{t}+\beta_{4}\left(Endline_{t}\times CO_{it}\right)+\beta_{5}\left(Endline_{t}\times CO_{it}^{2}\right)+\beta_{6}Treated_{i}+\beta_{7}\left(CO_{it}\times Treated_{i}\right)+\beta_{8}\left(CO_{it}^{2}\times Treated_{i}\right)+\beta_{9}\left(Endline_{t}\times Treated_{i}\right)+\beta_{10}\left(Endline_{t}\times CO_{it}\times Treated_{i}\right)+\beta_{11}\left(Endline_{t}\times CO_{it}^{2}\times Treated_{i}\right)+\varepsilon_{it}$, and test for the joint significance of *β*_9_, *β*_10_, and *β*_11_. The test is insignificant with *p* − *value* = 0.638, indicating that reporting did not change differently for treatment and control from the baseline to the end line. We also repeat this for both treatment groups separately and find similar results.

^17^
Levene's test of equality showed no statistically significant difference in the error variance, and residuals were normally distributed. Baseline and end line autocorrelation of outcomes is <0.40.

^18^
It is worth re‐emphasizing that the information participants recorded themselves in their logbook, as part of the logbook intervention, was not used for analysis since it could lead to potential measurement error being correlated with treatment assignment. Furthermore, comparable information does not exist for the control group. Instead, data collected during the baseline and end line surveys were used.

^19^
Comparable double‐difference estimates with *char*‐FE, and household‐FE for Tables 2, 4 and 5 are provided in Appendix Tables A3, A4, and A5, respectively, as robustness checks. Our results remain consistent. Results are also consistent when including household size, household consumption quintile, land ownership, age, education, the symptom of lung disease, agricultural worker, employer‐provided tobacco, shocks, and anti‐tobacco awareness campaigns exposure (in past 12 months) as controls. However, the inclusion of covariates may bias treatment effects in the absence of fully saturated regression models (Freedman, 2008). Therefore, we use a parsimonious specification throughout.

^20^
Since the dependent variable in this specification is the log of tobacco consumption expenditure, we follow (Wooldridge, 2015, p. 183–184) to get the accurate percentage change as $=\left[\exp\left(\hat{\beta }\right)-1\right]\times 100$.

^21^
Placebo regressions show that the interventions, as expected, did not have an effect on employer‐provided tobacco. Appendix Table A7 reports the results.

^22^
Table 3 consists of responses from male participants only. Female participants were not asked these questions due to an unfortunate oversight in the questionnaire design.

^23^
Despite statistically significant results in Tables 2 and 4, the interventions did not decrease smoking at a number of venues. One possible reason for this is the large standard errors. It might have been more difficult to recall tobacco usage at each of the several venues than to recall overall tobacco consumption. Further, questions about smoking behavior at different venues take binary responses only (extensive margin of smoking). The response to these questions would not have changed for someone who reduced smoking at one of these venues (intensive margin) but did not quit smoking at that venue completely. This further limits our ability to detect any effects on the smoking intensity at different locations.

^24^
It is worthwhile to note that the treatment effects on (male) smoking expenditure are much larger, −24.5% and −21.1% for the logbook and the poster interventions, respectively than those on (male) Breath CO Levels, −10% and −8.7% for the logbook and the poster treatments, respectively, reported in column (1) of Table 2. This is consistent with Figure A7 that shows that reductions in cigarette sticks have a small effect on CO when the consumption level of cigarette or *bidi* sticks is high. Panel B of Table 1 shows that the mean consumption was around 20 sticks. So looking again at Figure A7, we expect a smaller reduction in CO than in smoking because of the non‐linear dose‐response relationship.

^25^
At the end line, participants were also asked if maintaining the logbook encouraged them to consume less tobacco. Approximately 21% of logbook participants (*n* = 87) felt encouraged to consume less tobacco. This is, however, mostly driven by male participants (26% of male participants (*n* = 73) and only 11% (*n* = 14) of female participants felt encouraged). The association results, reported in Panel B of Appendix Table A6, indicates that male participants who reported feeling encouraged to consume less tobacco did in fact lower their smoking tobacco intake (but not SLT).

^26^
In a separate set of regressions, we find that the interventions are positively associated, although statistically insignificant, with a switch from expensive to less‐expensive smoking tobacco.

^27^
Sarkar et al. (2017), in a study conducted in Delhi, India, report a cost of less than £10 (∼USD 13) per treated tobacco user for a community intervention consisting of a single‐quit advice session (of 15 min) plus a single training session in yogic breathing exercises. Feenstra et al. (2005) examine the cost‐effectiveness for face‐to‐face smoking cessation interventions in The Netherlands and report estimated intervention costs per quit attempt of around €12 (∼USD 13) for minimal GP counseling, €35 (∼USD 38) for telephone counseling, €44 (∼USD 48) for minimal GP counseling with nicotine replacement therapy (NRT), and €243 for intensive counseling with NRT, among others. Our interventions are relatively cost‐effective.

^28^
Note that these calculations, $\frac{22.8}{4.9}\times 10=46.5\%$ and $\frac{25.3}{4.9}\times 10=51.6\%$, rely on the short‐term effects we estimate and assume linearity in the effect changing tobacco prices. One potential reason why we find larger effects is the shorter time lapse between our intervention and the end‐line survey. We expect the estimated effects would fall in the longer term, similar to Sarkar et al. (2017). Furthermore, this simplification ignores the likely substitution towards cheaper tobacco products that is expected if a multi‐tiered tax is used to increase tobacco prices.

^29^
Unfortunately, we could not find comparable studies that calculated the disability‐adjusted reduction in life years from smoking for Bangladesh. The closest study we could find was Efroymson et al. (2001). The authors calculate that if Bangladeshi households reallocated the money they spent on cigarettes to food, a typical poor smoker could add over 500 calories to the diet of one or two children in the household. They estimate that 10.5 million malnourished citizens could have an adequate diet and the lives of 350 children could be saved each day. Assuming these numbers have not changed significantly in the 20  years since the study, a 20%–25% decrease in a household's tobacco consumption that we find may have one‐fifth to one‐fourth of the potential beneficial effects they claim.

^30^
It is possible that we are unable to detect the small magnitude due to the low power of female‐only outcomes.

^31^
It is perhaps worthwhile to note that paying heed to the warning of “cherry‐picking” outcomes by Casey et al. (2012), we do not conduct the heterogeneity analysis using any outcome variables not specified in the pre‐analysis plan. Instead, we limit the exploration by participant characteristics at baseline to better understand potential mechanisms.

^32^
Admittedly, median splits are not the best statistical practice (Rucker et al., 2015). But it is widely accepted in the literature because of the clearer interpretations it offers in cases where the data approximates conceptual binary states (Iacobucci et al., 2015a,b).

^33^
Heterogeneous treatment effects for female participants are mostly insignificant with small magnitudes.

^34^
Appendix Table A10 reports the ex‐ante minimum detectable size (MDE) and power calculations. While ANCOVA helps to reduce the error variance and increase the power for detecting treatment effects (Haushofer & Shapiro, 2016; Maxwell et al., 2017; Shieh, 2017; van Breukelen, 2013), a larger number of clusters would have been ideal.

^35^
For example, Sarkar et al. (2017) report that the effectiftableveness of their intervention fell from 9.3% to 2% between 4‐weeks and 6‐months follow‐up.

^36^
The success of the Oral Therapy Education Program (OTEP) program by BRAC is one such example (Cash, 2021).

## Appendix A

**TABLE A1:** Price breakdown of commonly available tobacco products in the *chars* of Gaibandha

Brand name	Local price (in BDT, Dec 2018)	Quantity
Cigarettes		
Navy	50	Pack of 10 sticks
Merise	30	Pack of 10 sticks
Darby	30	Pack of 10 sticks
*Bidis*		
Aziz	15	Pack of 25 sticks
Akij	8	Pack of 25 sticks
Ashik	6	Pack of 25 sticks
Jonota	6	Pack of 25 sticks
*Zarda*		
Hapipuri	2	5 g
Baba	10	20 g
Shova	20	50 g
*Gul*		
Fancy	5	10 g

*Note*: *Bidis* are filter‐less locally produced smoking tobacco, and are cheaper alternatives to cigarettes. *Zarda* and *gul* are locally produced smokeless forms of tobacco. *Zarda* is normally chewed with betel leaf and areca nut, while *gul* is applied to the teeth and gums.

**TABLE A2:** Descriptive statistics and construction summary of additional variables

Variable	Construction	Mean
Lung damage	= 1 if predicted FEV1 value < 60, 0 otherwise.	0.424 (0.494)
	**Basis for the cut‐off:** We compared the FEV1 readings with the reference values for south East Asian males obtained from Quanjer et al. (2012). Using the cut‐off values by gender, age, and height we categorized the level of lung damage severity as mild (>70), moderate (60–69), moderately severe (50–59), severe (35–49), and very severe (<35). Following Pellegrino et al. (2005), we then generate a binary variable to identify those with moderately severe or worse lung damage.	
	**Rationale for variable selection:** Smoking is known to lead to a decline in FEV1 (Xu et al., 1994). FEV1 is an indicator of identifying chronic obstructive pulmonary disease (COPD) and lung damage (Clark et al., 1998). We wish to see if those with worse lung function react more to the interventions out of subjective health concern.	
Risk averse	= 1 if the predicted probability of making a safe choice is >0.50, 0 otherwise.	0.454 (0.498)
	**Basis for the cut‐off:** Previous literature on the topic generally considers participants with less than 50% probability of making a safe choice as risk‐averse (Fakir, 2021; Harrison et al., 2017).	
	**Rationale for variable selection:** There is a rich theoretical literature in the economics of habit formation that posits that preferences may differ between addicts and non‐addicts (Harrison et al., 2018). We wish to see if this mediates the impact of the intervention.	
Patience	= 1 if a participant switches from a smaller immediate reward to a larger delayed reward (1 month) for a discount rate of *r* ≤ 0.15 (survey sample median), 0 otherwise.	0.516 (0.497)
	**Basis for the cut‐off:** Those with a lower discount rate are considered more patient than those with a higher discount rate. We use the survey sample median discount rate to segregate the sample.	
	**Rationale for variable selection:** Besides the theoretical literature on the importance of time preference in explaining smoking Becker and Murphy (1988), there is also a large body of empirical evidence that documents the correlations between discount rates and smoking (Chabris et al., 2008; Jung et al., 2021). More patient participants are likely respond better to the interventions.	
No shock	= 1 for participants who did not face any shocks in the past 12 months, 0 otherwise.	0.380 (0.486)
	**Basis for the cut‐off:** Shocks are often used as a proxy for stress. Since hardly anyone reported experiencing more than one shock in the 12 months prior to the baseline survey, it was logical to generate a dummy variable that identified exposure to any shocks.	
	**Rationale for variable selection:** Studies have found smoking is often used as a coping mechanism during stressful periods (Everding & Marcus, 2020). Participants with less stress may thus be more amenable to the interventions' messages.	
Relocation ≤5	= 1 for participants with ≤5 lifetime *char* re‐locations (survey sample median), 0 otherwise.	0.507 (0.491)
	**Basis for the cut‐off:** Households in the *chars* often have to relocate to other *chars* due to land erosion or eviction. Repeated re‐locations thus act as a proxy for *long‐term* stress on the household participants. We use the survey sample median to segregate the sample.	
	**Rationale for variable selection:** Same as under “no shock.”	
Tobacco exp >5.5%	= 1 for participants who spent >5.5% (survey sample median) of household consumption expenditure behind tobacco, 0 otherwise.	0.492 (0.500)
	**Basis for the cut‐off:** We segregate the sample at the survey sample median of 5.5% (instead of the mean of 9%).	
	**Rationale for variable selection:** If the interventions operate through greater cost‐saving incentives, participants who spend a larger share of their household consumption expenditure behind tobacco may respond more.	
Child <5	= 1 for presence of children below the age of five in participant's household, 0 otherwise.	0.345 (0.476)
	**Basis for the cut‐off:** Children below the age of five are more likely to stay home most of the day without any school obligations. They are more likely to be exposed to second‐hand smoke.	
	**Rationale for variable selection:** If the interventions operate through a sense of altruism towards children (Hunter et al., 2020), participants who have children living with them in the household may respond more (Park & Kim, 2018).	
Any schooling	= 1 if participant has no schooling, 0 otherwise.	0.380 (0.486)
	**Basis for the cut‐off:** It would have been ideal to segregate the sample at the median education level. However, since more than half of the participants did not have any schooling, we segregate by whether participants had any of schooling.	
	**Rationale for variable selection:** Educated individuals are less likely to smoke (De Walque, 2007). They may be more likely to internalize associated health costs better than those without any schooling, and thus may respond more to the interventions.	

*Note*: Variable mean reported at baseline (standard deviation in parenthesis) for the combined sample of male and female participants, except for “Risk Averse” and “Patience”, where the information was collected only for male participants.

**TABLE A3:** Treatment effects on CO level and household tobacco expenditure (Double difference estimates)

	(1)	(2)	(3)	(4)
	Breath CO level	Total tobacco expenditure (*log*)	Breath CO level	Expenditure (*log*)
Post × logbook intervention (LT)	−0.327**	−0.218***	−0.331**	−0.232***
	(0.153)	(0.075)	(0.157)	(0.072)
Post × poster intervention (PT)	−0.250***	−0.225**	−0.250**	−0.211***
	(0.089)	(0.072)	(0.118)	(0.068)
FWER *p*‐value (LT)	0.043	0.000	0.045	0.000
FWER *p*‐value (PT)	0.029	0.000	0.031	0.000
CGM *p*‐value (LT)	0.007	0.000	0.007	0.000
CGM *p*‐value (PT)	0.021	0.000	0.022	0.000
RI *p*‐value (LT)	0.012	0.000	0.013	0.000
RI *p*‐value (PT)	0.033	0.000	0.032	0.000
LT=PT *p*‐value	0.664	0.946	0.680	0.832
Char (FE)	Yes	Yes	No	No
Household FE	No	No	Yes	Yes
Control mean of DV	4.391	2.557	4.391	2.557
Observations	1654	1718	1654	1718

*Note*: Coefficient are double difference estimates. Robust standard errors clustered at the char‐level are in parentheses. “Total Tobacco Expenditure (log)” in columns (2) and (4) measures the sum of tobacco use for the two partners while the “Breath CO Level” in columns (1) and (3) is for males only (since none of the females smoked). Total tobacco expenditure are the log of winsorized (at the 99% level) daily values in BDT. Breath CO levels are in 7 discrete categories of 0–6, 7–10, 11–15, 16–20, 21–25, 26–30, and 31+ parts per million (ppm). CGM *p*‐values are calculated using the wild bootstrap‐t clustering method (Cameron et al., 2008). RI *p*‐values are the Alwyn Young randomization inference based *p*‐values with 1000 replications (Young, 2019).

Abbreviations: CO, carbon‐monoxide; DV, dependent variable; FE, fixed effects; FWER, family‐wise error rate; LT, Logbook intervention; PT, poster intervention, RI, randomization inference.

****p* < 0.01, ***p* < 0.05, **p* < 0.1.

**TABLE A4:** Treatment effects by type of tobacco consumption (Double difference estimates)

	(1)	(2)	(3)	(4)	(5)	(6)	(7)	(8)	(9)	(10)
	Smoking tobacco	Cigarette sticks	Bidi sticks	Smokeless tobacco	Smokeless frequency	Smoking tobacco	Cigarette sticks	Bidi sticks	Smokeless tobacco	Smokeless frequency
Variables	Expenditure			Expenditure		Expenditure			Expenditure	
Post × logbook treatment (LT)	−0.221***	−2.352***	−0.628	0.173**	0.502**	−0.217***	−2.465***	−0.661	0.181**	0.521**
	(0.075)	(0.893)	(0.528)	(0.071)	(0.223)	(0.055)	(0.449)	(0.520)	(0.073)	(0.210)
Post × poster treatment (PT)	−0.206***	−1.952***	−2.265*	−0.113	−0.346	−0.203***	−1.956***	−2.371*	−0.116	−0.358
	(0.072)	(0.695)	(1.292)	(0.079)	(0.227)	(0.041)	(0.428)	(1.127)	(0.096)	(0.253)
FWER *p*‐value (LT)	0.000	0.000	0.386	0.054	0.048	0.000	0.000	0.354	0.045	0.049
FWER *p*‐value (PT)	0.000	0.000	0.051	0.198	0.143	0.000	0.000	0.054	0.229	0.152
CGM *p*‐value (LT)	0.000	0.000	0.251	0.037	0.028	0.000	0.000	0.212	0.021	0.031
CGM *p*‐value (PT)	0.000	0.000	0.073	0.155	0.167	0.000	0.000	0.068	0.187	0.168
RI *p*‐value (LT)	0.000	0.000	0.248	0.046	0.022	0.000	0.000	0.231	0.023	0.025
RI *p*‐value (PT)	0.000	0.000	0.067	0.187	0.185	0.000	0.000	0.080	0.201	0.188
LT=PT *p*‐value	0.885	0.724	0.241	0.007	0.007	0.838	0.412	0.168	0.014	0.008
Char FE	Yes	Yes	Yes	Yes	Yes	No	No	No	No	No
Household FE	No	No	No	No	No	Yes	Yes	Yes	Yes	Yes
Control mean of DV	2.223	5.735	13.557	1.376	2.251	2.223	5.735	13.557	1.376	2.251
Observations	1654	1654	1654	1718	1718	1654	1654	1654	1718	1718

*Note*: Coefficient are double difference estimates. Robust standard errors clustered at the char‐level are in parentheses. Since only males smoked, “Smoking Tobacco Exp. *(log)*,” “Cigarette Sticks,” and “Bidi Sticks” in columns (1)‐(3) and (6)‐(8) reflect expenditure behind smoking tobacco by males only. “Smokeless Tobacco Exp. *(log)*” and “Smokeless Frequency” in columns (4)‐(5) and (9)‐(10) is the sum for the two partners. Expenditure values in columns (1), (4), (6), and (9) are the log of winsorized (at the 99% level) daily values in BDT; stick values in columns (2), (3), (7), and (8), and frequency values in columns (5) and (10), are counts of respective daily intake. CGM *p*‐values are calculated using the wild bootstrap‐t clustering method (Cameron et al., 2008). RI *p*‐values are the Alwyn Young randomization inference based *p*‐values with 1000 replications (Young, 2019).

Abbreviations: DV, dependent variable; FE, fixed effects; FWER, family‐wise error rate; LT, Logbook intervention; PT, poster intervention, RI, randomization inference.

****p* < 0.01, ***p* < 0.05, **p* < 0.1.

**TABLE A5:** Treatment effects on SLT consumption by gender (Double difference estimates)

	(1)	(2)	(3)	(4)	(5)	(6)	(7)	(8)
	Smokeless tobacco expenditure	Smokeless frequency	Smokeless tobacco expenditure	Smokeless frequency	Smokeless tobacco expenditure	Smokeless frequency	Smokeless tobacco expenditure	Smokeless frequency
Variables	Males				Females			
Post × logbook treatment (LT)	0.179**	0.656**	0.163*	0.568**	−0.091	−0.188	−0.064	−0.165
	(0.077)	(0.296)	(0.075)	(0.254)	(0.066)	(0.141)	(0.059)	(0.131)
Post × poster treatment (PT)	−0.017	−0.109	−0.016	−0.097	−0.107*	−0.249*	−0.099*	−0.217*
	(0.075)	(0.219)	(0.078)	(0.186)	(0.063)	(0.132)	(0.056)	(0.115)
FWER *p*‐value (LT)	0.035	0.041	0.046	0.040	0.391	0.426	0.611	0.443
FWER *p*‐value (PT)	0.717	0.782	0.811	0.770	0.079	0.080	0.083	0.080
CGM *p*‐value (LT)	0.027	0.034	0.042	0.031	0.368	0.405	0.575	0.415
CGM *p*‐value (PT)	0.681	0.771	0.723	0.756	0.068	0.071	0.080	0.070
RI *p*‐value (LT)	0.022	0.029	0.038	0.028	0.355	0.411	0.562	0.403
RI *p*‐value (PT)	0.675	0.720	0.701	0.698	0.065	0.068	0.079	0.069
LT=PT *p*‐value	0.068	0.038	0.098	0.035	0.861	0.752	0.667	0.766
Char FE	Yes	Yes	No	No	Yes	Yes	No	No
Household FE	No	No	Yes	Yes	No	No	Yes	Yes
Control mean of DV	0.803	1.433	0.803	1.433	1.460	3.526	1.460	3.526
Observations	1654	1654	1654	1654	776	776	776	776

*Note*: Coefficient are double difference estimates. Robust standard errors clustered at the char‐level are in parentheses. Expenditure values in columns (1), (3), (5), and (7) are the log of winsorized (at the 99% level) daily values in BDT; frequency values in columns (2), (4), (6), and (8) are counts of daily intake. CGM *p*‐values are calculated using the wild bootstrap‐t clustering method (Cameron et al., (2008). RI *p*‐values are the Alwyn Young randomization inference based *p*‐values with 1000 replications (Young, 2019).

Abbreviations: DV, dependent variable; FE, fixed effects; FWER, family‐wise error rate; LT, Logbook intervention; PT, poster intervention, RI, randomization inference; SLT, smokeless tobocco.

****p* < 0.01, ***p* < 0.05, **p* < 0.1.

**TABLE A6:** Associations of compliance with logbook treatment

	(1)	(2)	(3)	(4)	(5)
	Household		Males		Females
	Total tobacco exp. *(log)*	CO level	Smoking tobacco exp. *(log)*	Smokeless tobacco exp. *(log)*	Smokeless tobacco exp. *(log)*
**Panel A: Logbook entry**					
Daily logbook entry	−0.179	−0.219	−0.145	0.083	−0.074
	(0.285)	(0.454)	(0.144)	(0.142)	(0.198)
**Panel B: Encouraged to reduce tobacco intake**					
Encouraged to reduce	−0.381**	−0.171*	−0.355**	0.082	−0.066
	(0.198)	(0.102)	(0.134)	(0.182)	(0.080)
Controls	Yes	Yes	Yes	Yes	Yes
Char FE	Yes	Yes	Yes	Yes	Yes
Mean of DV	2.505	4.308	2.065	0.915	1.355
Observations	292	284	284	284	131

*Note*: Coefficients are ordinary least squares regression estimates. Robust standard errors clustered at the *char*‐level are in parentheses. “Total Tobacco Expenditure (log)” in column (1) measures the sum of tobacco use for the two partners. Expenditure values in columns (1), (3), (4), and (5) are the log of winsorized (at the 99% level) daily values in BDT. Breath CO levels in column (2) are measured in 7 discrete categories of 0–6, 7–10, 11–15, 16–20, 21–25, 26–30, and 31+ parts per million (ppm). During end line, enumerators checked if participants in the logbook treatment made entries for each day and asked participants about their frequency of logbook entry (daily, weekly, or not at all). About 77% of participants reported making daily entries, while the remaining 23% had made entries weekly. In Panel A, Daily Logbook Entry = 1 if daily entries were made, and = 0 otherwise. Participants were also asked if maintaining the logbook encouraged them to consume less tobacco. Approximately 21% participants (*n* = 87) felt encouraged to consume less tobacco. In Panel B, Cessation Encouragement = 1 if participant reported feeling encouraged to reduce intake, and = 0 otherwise. Controls include household size, household consumption quintile, land ownership, age, agricultural worker, employer‐provided tobacco, and anti‐tobacco awareness campaigns exposure (in past 12 months).

Abbreviations: CO, carbon‐monoxide; DV, dependent variable; FE, fixed effects.

****p* < 0.01, ***p* < 0.05, **p* < 0.1.

**TABLE A7:** Placebo regression on employer provided tobacco

	(1)	(2)
	Employer provides free tobacco (=1)	Monetary value of employer provided tobacco *(log)*
Logbook intervention (LT)	0.025	0.049
	(0.067)	(0.081)
Poster intervention (PT)	0.020	−0.042
	(0.072)	(0.070)
LT=PT *p*‐value	0.959	0.396
Control mean of DV	0.465	1.244
Observations	827	385

*Note*: Coefficients are intent‐to‐treat estimates. Robust standard errors clustered at the *char*‐level are in parentheses. All regressions control for the baseline value of their respective outcomes. Regressions run for male participants only since less than 2% of female participants receive employer provided tobacco. Monetary value in column (2) is the log of winsorized (at the 99% level) daily values in BDT.

Abbreviations: DV, dependent variable; FLT, Logbook intervention; PT, poster intervention.

****p* < 0.01, ***p* < 0.05, **p* < 0.1.

**TABLE A8:** Associations of poster intervention emotional reactions

	(1)	(2)	(3)	(4)	(5)
	Sad	Guilt	Shame	Fear	Disgust
Lung damage	−0.002	0.046*	0.044	−0.002	0.009
	(0.009)	(0.025)	(0.028)	(0.020)	(0.018)
Risk averse	−0.013	−0.012	−0.002	−0.007	−0.009
	(0.019)	(0.013)	(0.012)	(0.015)	(0.015)
No shock	−0.025	−0.047	−0.027	−0.030	−0.018
	(0.059)	(0.043)	(0.026)	(0.071)	(0.061)
Relocation ≤5	−0.013	0.002	0.012	0.019	0.026
	(0.021)	(0.016)	(0.027)	(0.022)	(0.020)
Tobacco exp >5.5%	−0.012	0.005	0.022	0.041	0.011
	(0.018)	(0.031)	(0.025)	(0.028)	(0.033)
Child <5 years	0.045*	0.004	0.040**	0.066*	0.053**
	(0.023)	(0.026)	(0.019)	(0.037)	(0.024)
Any schooling	0.000	0.005	0.027**	0.004	0.019
	(0.021)	(0.020)	(0.013)	(0.024)	(0.015)
Patience (time pref.)	0.023	0.056**	0.012	0.019	0.017
	(0.020)	(0.026)	(0.019)	(0.035)	(0.031)
Controls	Yes	Yes	Yes	Yes	Yes
Char FE	Yes	Yes	Yes	Yes	Yes
Mean of DV	0.044	0.779	0.717	0.773	0.727
Observations	287	287	287	287	287

*Note*: Coefficients are ordinary least squares regression estimates. Male participants were asked about their emotional reactions to the posters at end line. Emotional reactions were assessed on a 7‐point ordinal Likert scale (with values from 1 to 7) ranging from “strongly disagree” to “strongly agree.” Binary variables were then constructed where respective emotions were coded as = 1 if participants' ranked ≥5 on the Likert scale. The construction of indicator variables is explained in Table A2. Controls include household size, household consumption quintile, land ownership, age, agricultural worker, employer‐provided tobacco, and anti‐tobacco awareness campaigns exposure (in past 12 months).

Abbreviations: DV, dependent variable; FE, fixed effects.

****p* < 0.01, ***p* < 0.05, **p* < 0.1.

**TABLE A9:** Heterogeneity in the impact on total tobacco expenditure (Male participants)

	Total tobacco expenditure (*log*)								
	(1)	(2)	(3)	(4)	(5)	(6)	(7)	(8)	(9)
Logbook treatment (LT)	−0.170^++^	−0.137^+++^	−0.212^++^	−0.199^+++^	−0.104^++^	−0.203^++^	−0.170^+^	−0.185^+^	−0.127^+^
	(0.067)	(0.042)	(0.094)	(0.037)	(0.048)	(0.075)	(0.081)	(0.101)	(0.064)
Poster treatment (PT)	−0.220^+^	−0.262^++^	−0.179^+^	−0.242^+^	−0.137^+^	−0.194^+++^	−0.223^++^	−0.207^++^	−0.149^++^
	(0.105)	(0.121)	(0.087)	(0.117)	(0.085)	(0.061)	(0.093)	(0.098)	(0.068)
LT × Lung damage	−0.031								−0.032
	(0.212)								(0.252)
PT × Lung damage	−0.025								−0.037
	(0.253)								(0.272)
LT × Risk averse		−0.175^+^							−0.166
		(0.084)							(0.098)
PT × Risk averse		0.098							0.130
		(0.235)							(0.337)
LT × No shock			−0.283						−0.174
			(0.382)						(0.444)
PT × No shock			−0.309						−0.326
			(0.355)						(0.362)
LT × Relocation ≤5				−0.226^+++^					−0.182^+^
				(0.081)					(0.090)
PT × Relocation ≤5				−0.013					−0.062
				(0.225)					(0.242)
LT × Tobacco exp >5.5%					−0.211^+^				−0.289
					(0.117)				(0.170)
PT × Tobacco exp >5.5%					−0.197				−0.207
					(0.234)				(0.255)
LT × Child <5 years						−0.175			−0.095
						(0.254)			(0.243)
PT × Child <5 years						−0.276^++^			−0.314^++^
						(0.120)			(0.139)
LT × Any schooling							−0.203		−0.141
							(0.215)		(0.257)
PT × Any schooling							−0.154^++^		−0.115^+++^
							(0.072)		(0.040)
LT × Patience (time pref.)								−0.087	−0.007
								(0.210)	(0.215)
PT × Patience (time pref.)								−0.186^++^	−0.155
								(0.077)	(0.120)

Overall effect on indicated category									
Logbook treatment (LT)	−0.202	−0.312**	−0.495	−0.425***	−0.315*	−0.378	−0.372*	−0.272	‐
	(0.170)	(0.166)	(0.357)	(0.158)	(0.173)	(0.236)	(0.222)	(0.198)	‐
Poster treatment (PT)	−0.245	−0.164	−0.489	−0.255	−0.334	−0.470***	−0.377***	−0.393**	‐
	(0.190)	(0.148)	(0.297)	(0.147)	(0.232)	(0.178)	(0.112)	(0.157)	‐
LT=PT *p*‐value	0.688	0.329	0.797	0.726	0.735	0.926	0.667	0.876	0.814
LT=PT interaction *p*‐value	0.985	0.274	0.960	0.373	0.957	0.719	0.829	0.669	‐
Control mean of DV	2.361	2.361	2.361	2.361	2.361	2.361	2.361	2.361	2.361
Observations	827	827	827	827	827	827	827	827	827

*Note*: Family‐wise error rate (FWER) *p‐values*: ^+++^ *p* < 0.01, ^++^ *p* < 0.05, ^+^ *p* < 0.1. Robust standard error *p‐values*: ****p* < 0.01, ***p* < 0.05, **p* < 0.1. Coefficients are intent‐to‐treat estimates from fully saturated regressions. Robust standard errors clustered at the *char*‐level are in parentheses. All regressions control for the baseline value of their respective outcomes. Total tobacco expenditure values are the log of winsorized (at the 99% level) daily values in BDT. The construction of indicator variables is explained in Table A2.

Abbreviations: DV, dependent variable; LT, Logbook treatment; PT, posterior treatment.

**TABLE A10:** Ex‐ante MDE and power calculations

	Power		
Outcome	*δ* = 0.20	*δ* = 0.25	*δ* = 0.30
Household			
Breath CO level	0.704	0.860	0.948
Total tobacco expenditure	0.715	0.869	0.953
Smoking tobacco expenditure	0.730	0.880	0.959
Cigarette sticks	0.451	0.601	0.738
Bidi sticks	0.353	0.472	0.602
SLT expenditure	0.739	0.887	0.963
SLT frequency	0.316	0.426	0.541
Males			
SLT expenditure	0.755	0.898	0.968
SLT frequency	0.315	0.425	0.541
Females			
SLT expenditure	0.560	0.726	0.853
SLT frequency	0.372	0.501	0.630

*Note*: Ex‐ante calculations done based on experiment sample design and standard deviations from pilot in three out‐of‐sample *chars*. While a greater number of clusters would have helped to improve the power, we were restricted by the available experiment budget.

Abbreviations: CO, carbon‐monoxide; MDE, minimum detectable size; SLT, smokeless tobacco.
